# Identification and analysis of key genes involved in methyl salicylate biosynthesis in different birch species

**DOI:** 10.1371/journal.pone.0240246

**Published:** 2020-10-08

**Authors:** Kiran Singewar, Christian R. Moschner, Eberhard Hartung, Matthias Fladung

**Affiliations:** 1 Institute of Agricultural Process Engineering, Christian-Albrechts University of Kiel, Kiel, Schleswig-Holstein, Germany; 2 Thünen Institute of Forest Genetics, Grosshansdorf, Schleswig-Holstein, Germany; USDA-ARS Southeast Area, UNITED STATES

## Abstract

Species of the perennial woody plant genus *Betula* dominate subalpine forests and play a significant role in preserving biological diversity. In addition to their conventional benefits, birches synthesize a wide range of secondary metabolites having pharmacological significance. Methyl salicylate (MeSA) is one of these naturally occurring compounds constitutively produced by different birch species. MeSA is therapeutically important in human medicine for muscle injuries and joint pain. However, MeSA is now mainly produced synthetically due to a lack of information relating to MeSA biosynthesis and regulation. In this study, we performed a comprehensive bioinformatics analysis of two candidate genes mediating MeSA biosynthesis, *SALICYLIC ACID METHYLTRANSFERASE* (*SAMT*) and *SALICYLIC ACID-BINDING PROTEIN* 2 (*SABP2*), of high (*B*. *lenta*, *B*. *alleghaniensis*, *B*. *medwediewii*, and *B*. *grossa*) and low (*B*. *pendula*, *B*. *utilis*, *B*. *alnoides*, and *B*. *nana*) MeSA-producing birch species. Phylogenetic analyses of *SAMT* and *SABP2* genes and homologous genes from other plant species confirmed their evolutionary relationships. Multiple sequence alignments of the amino acid revealed the occurrence of important residues for substrate specificity in SAMT and SABP2. The analysis of *cis* elements in different birches indicated a functional multiplicity of *SAMT* and *SABP2* and provided insights into the regulation of both genes. We successfully developed six prominent single nucleotide substitution markers that were validated with 38 additional birch individuals to differentiate high and low MeSA-producing birch species. Relative tissue-specific expression analysis of *SAMT* in leaf and bark tissue of two high and two low MeSA-synthesizing birches revealed a high expression in the bark of both high MeSA-synthesizing birches. In contrast, *SABP2* expression in tissues revealed indifferent levels of expression between species belonging to the two groups. The comparative expression and bioinformatics analyses provided vital information that could be used to apply plant genetic engineering technology in the mass production of organic MeSA.

## Introduction

Methyl salicylate (MeSA) is a volatile compound, widespread in many plant species, that has been extensively studied as a long-distance mobile signaling molecule in systemic acquired resistance (SAR) [1]. SAR is an inducible defense mechanism, activated in response to pathogen attack [2]. Salicylic acid (SA) produces MeSA by the action of salicylic acid methyltransferase (SAMT) utilizing S-adenosyl-L-methionine (SAM) as a cofactor, which is the most widely used methyl donor for enzymatic methyl transfer reactions [3]. The SAMT enzyme accumulates at the site of infection [4] since the systematic collection of MeSA in the infected tissue is required for the successful functioning of SAR [5]. In contrast, the salicylic acid-binding protein 2 (SABP2) with strong esterase activity catalyzes the reaction of MeSA to SA synthesis [2, 6]. The overexpression of *SABP2* or silencing of *SAMT* reduces the accumulation of MeSA in the infected plant leaves, resulting in SAR depletion [7], indicating that *SAMT* and *SABP2* synchronize MeSA levels in plants [8]. High concentrations of SA, arising from the shikimate acid pathway, might be toxic to plants [9] and thus could be one reason for MeSA production [10].

Many studies have shown that plants produce MeSA following herbivore attacks in order to attract the natural enemies of these herbivores [11, 12]. The term for these compounds is herbivore-induced plant volatile (HIPV) [13]. Arthropods, the natural enemies of herbivores, are attracted by HIPVs and in this way increase biological control activity [14]. According to Coppola et al., [15], aphids behave differently on plants treated with MeSA compared to untreated plants. Aphids rapidly abandon MeSA-treated plants, confirming its direct impact on their dispersal. Considerable amounts of MeSA are emitted from plant vegetation into the environment [16]. Before emission, the volatile compound is stored in specialized glandular cells or organelles and can be released constitutively under stress, but also under optimal conditions [17]. Rigorous studies on SA-dependent MeSA production have been conducted on a variety of annual plant species, including rice [18], *Arabidopsis* [19, 20] and tobacco [21]. However, to the best of our knowledge, studies of constitutive MeSA production in plants are mostly absent in the literature.

The importance of MeSA as a natural phytochemical compound in therapeutics was acknowledged very early on in human history [22]. For the American and Canadian indigenous communities, plants were the main source of MeSA as a substance to reduce pain [22, 23]. The leaves and bark of birches were used as a basis for herbal infusions for the treatment of fever and gastrointestinal ailments [24, 25]. Commonly, MeSA is used as an essential oil and fragrance [26] and possesses an anti-inflammatory effect that has been used for pain relief and in many medicinal products for muscle and joint pain and rheumatic conditions [27]. From its original extraction through traditional herbal procedures, MeSA is now a mass-produced, synthetic and pharmaceutically significant substance [23]. The use of natural MeSA is limited due to an insufficient supply of the natural raw product, and the constructive utilization of natural plant resources is often critical. The limited distribution of the natural product in certain species, tissues and organs requires an improvement in the molecular and phytochemical knowledge relating to the product that is vital to the development of herbal medicine [28].

Several species of the genus *Betula* (specifically the subgenus *Aspera*, section *Lentae*) are among the examples that constitutively produce high levels of MeSA [22, 29, 30]. A chemical analysis of birch extract (bark and leaf) revealed a concentration of between 49–99.8% of MeSA in the extracted essential oil constituents [31–33]. Birches belong to one of the vital angiosperm genera that support and benefit thousands of living organisms, as well as maintain the ecosystem in boreal forests [30, 34, 35]. Apart from their medicinal and traditional uses, many species of this genus have a long history of difficulties in their classification [36–38]. Considering taxonomical issues, the classification of birches using molecular markers for high and low MeSA production is mostly absent in the literature.

New pharmaceutically significant and naturally existing substances, together with their therapeutic and regenerative features, are being constantly investigated. Therefore, bioinformatics and the expression analysis of genes involved in MeSA biosynthesis will be crucial for cloning and functional analysis studies. It is possible to regulate MeSA biosynthesis through genetic engineering or genome editing technologies.

In this study, we attempted to answer the question of whether, if any, there are common variations in the genes that contribute to an increased MeSA content in some *Betula* species. Thus, we targeted (1) the intra and interspecific comparative bioinformatics analysis of candidate genes in different low and high MeSA-producing *Betula* species, (2) the sequence variation analysis and marker development in candidate genes associated with MeSA production and (3) the relative expression analysis of two candidate genes that mediate MeSA biosynthesis in high and low MeSA-producing *Betula* species from two different subgenera.

To achieve this, we analyzed *SAMT* and *SABP2* candidate genes that mediate MeSA biosynthesis in eight *Betula* species. The sequencing analysis of *SAMT* and *SABP2* genes revealed putative nucleotide variation associated with high and low MeSA production in birches. The tissue-specific expression analyses of the candidate genes showed differential expression in the *Betula* species.

## Materials and methods

### Plant material

The seeds of eight *Betula* species (*B*. *alleghaniensis*, *B*. *alnoides*, *B*. *lenta*, *B*. *grossa*, *B*. *medwediewii*, *B*. *pendula* and *B*. *utilis*) were collected from different botanical gardens in Germany. Specific permissions for the sample collection were obtained from the authority responsible for respective botanical gardens. Seed germination was carried out in standard soil with 10–30% humidity and pH 6.5 in a natural environment without any fertilizer in a polyhouse at the Institute of Agricultural Process Engineering, Kiel University, Germany. Plantlet cultivation was implemented with the required dose of fertilizers in a glasshouse under natural daylight conditions at the Thünen-Institute of Forest Genetics, Grosshansdorf, Germany. The birch species previously confirmed through barcoding and phylogenetic analysis [29] were selected for this study (Table 1).

**Table 1:** Details of the species used for *SAMT* and *SABP2* candidate gene analyses: Names of the species, place of sample origin, geographical distribution, ploidy levels and taxonomic positions were allocated according to Wang et al. (2016) and Ashburner and McAllister (2013).

Species Name	Individuals*	Physical origin	Distribution	2n	Subgenus	Section
*B*. *alleghaniensis*	6	BG Tharandt, Germany	North America	6n	*Aspera*	*Lentae*
*B*. *lenta*	6	BG Giessen, Germany	North America	2n	*Aspera*	*Lentae*
*B*. *medwediewii*	4	BG Tharandt, Germany	Japan	2n	*Aspera*	*Lentae*
*B*. *grossa*	4	BG Tharandt, Germany	Japan	12n	*Aspera*	*Lentae*
*B*. *alnoides*	4	BG Tharandt, Germany	India, Bhutan, Nepal	2n	*Acuminata*	*Acuminatae*
*B*. *pendula*	6	BG Grosshansdorf, Germany	Europe and East Asia	2n	*Betula*	*Betula*
*B*. *utilis*	4	Kiel (private), Germany	Himalayas	4n	*Betula*	*Costatae*
*B*. *nana*	4	BG Cambridge, England	Arctic region	2n	*Betula*	*Apterocaryon*

*Selected number of individuals per species.

MeSA-producing ability was classified according to monographic descriptions [22, 30–32] and own analytical evidence, i.e. olfactory analysis following the scratching of the bark [29]: *B*. *alleghaniensis*, *B*. *medwediewii*, *B*. *grossa*, and *B*. *lenta* (subgenus: *Aspera*) were classified as high MeSA producers, while *B*. *pendula*, *B*. *alnoides*, *B*. *uitilis*, and *B*. *nana* (subgenus: *Betula*) were classified as low MeSA producers.

### Identification of candidate genes and retrieval of sequences

The previously functionally characterized protein sequence of *Clarkia breweri* SAMT (accession number: AF133053) [4] and *Nicotiana tabacum* SABP2 (accession number: AY485932) [7] were used as queries in the silver birch (*B*. *pendula*) genome database (https://genomevolution.org/coge/CoGeBlast.pl) to search for homologous sequences using the BlastP algorithm [39]. The silver birch candidate genes encoding SAMT and SABP2 proteins were selected according to the highest-scoring pair and E-value after the Blast search [39] (S1 Table in S1 File). Additionally, the phylogenetic closeness of the *B*. *pendula* candidate genes with the references *CbSAMT* (S1 Fig in S1 File) and *NtSABP2* (S2 Fig in S1 File), were also considered as selection criteria. Thirty-two SAMT and thirteen SABP2 previously identified protein sequences from different plant species were retrieved from the NCBI (https://www.ncbi.nlm.nih.gov/) and PopGenIE (http://popgenie.org/) gene databases for phylogenetic analysis (S2 Table in S1 File).

### DNA extraction, amplification and sequence analysis

Total DNA was extracted from the leaves of the plants according to the CTAB protocol [40]. DNA extraction of *B*. *medwediewii*, *B*. *alleghaniensis*, and *B*. *lenta* was difficult due to the presence of a high level of polysaccharides, therefore a pre-washing buffer [41] (1.6 ml ice-cold TNE buffer: 200 mM Tris-HCl, 250 mM NaCl, 50 mM EDTA) was used to extract a sufficient quality of DNA.

Specific primers were designed (S3 Table in S1 File) based on the respective *B*. *pendula* gene sequences [42] as references for amplifying the exon and promoter regions of the *SABP2* and *SAMT* genes of the different birch species (Table 1) using polymerase chain reaction (PCR). The following cycling conditions were used for the PCR amplification: 95°C for 3 min, 40 cycles at 95°C for 10 s, 60°C for 30 s and 72°C for 30 s. All PCR reactions were performed in a SensoQuest thermocycler (Göttingen, Germany). The PCR products were confirmed on 1% agarose gel stained with Roti^®^GelStain (Carl Roth, Germany). The StarSEQ (Mainz, Germany) service was used for sequencing.

Electropherograms of each sequence were visually inspected. All sequences were aligned and screened for the presence of polymorphism using the SeqManPro 15 program from the DNASTAR Lasergene bioinformatics software suite (Madison, Wisconsin USA).

### Multiple sequence alignment and phylogenetic analysis

The retrieved protein sequences from the NCBI (https://www.ncbi.nlm.nih.gov/) and PopGenIE (http://popgenie.org/) gene databases were aligned using ClustalW with default parameters and maximum likelihood (ML) trees were constructed using MEGA X [43] with a bootstrap value of 1,000 replicates. The MES *Beauveria bassiana* (PMB68924.1) and SAMT *Aspergillus niger* (NT166520) protein sequences were used as the outgroup species in the phylogenetic analysis. Additionally, two more (S4A and S4B Fig in S1 File) phylogenies were constructed using eight birch species to analyze intraspecific relationships. The DNA sequences from the birch *SAMT* and *SABP2* were translated into amino acid sequences using the ExPASy translation tool (https://web.expasy.org/translate/).

### Gene structure, conserved domain, motif and promoter analysis

The intron/exon organization of the *SAMT* and *SABP2* genes of *B*. *pendula* were predicted based on the respective genomic and coding DNA sequences retrieved from the available *B*. *pendula* genome (https://genomevolution.org/coge/CoGeBlast.pl). The conserved domains were analyzed using the motif online search tool (https://www.genome.jp/tools/motif/). The conserved motifs in the *Betula* proteins were identified using the MEME online tool (http://meme-suite.org/tools/meme) with the following parameters: maximum number of motifs, 11; minimum motif width, 6 and maximum motif width, 60. The promoter region of the *SAMT* and*SABP2* genes was examined in *B*. *pendula* and other *Betula* species under investigation using the option “search for care” in the PlantCARE database [44].

### RNA extraction and RT-qPCR

The leaf and bark tissues of three-year-old plants were harvested on the morning of 8 July 2019 from the four different *Betula* species, including *B*. *alleghaniensis*, *B*. *lenta*, *B*. *pendula* and *B*. *utilis* (three biological replicates per species). Total RNA was extracted from the leaf and bark of the plants using the Spectrum Plant Total RNA Kit manufactured by Sigma-Aldrich. Extracted RNA was treated with Invitroge TURBO DNA- free Kit (Thermofisher Scientific, Dreieich, Germany) according to the manufacturer’s instructions to remove residual DNA before the next steps. RNA samples were selected based on the rRNA band intensities (28S/18S = 2:1) with a Nanodrop spectrometer (Thermo Scientific, Germany), with optical density values A260 nm/A280 nm between 1.8–2.0 absorption ratio, and A260 nm/A230 nm higher than 1.8 absorption ratio. The first-strand of cDNA was synthesized using GoScrip Reverse Transcription Mix, Oligo(dT) (Promega, Germany). The reverse transcription reaction included 10 μl RNA, 4 μl reaction buffer, 2 μl GoScript Enzyme, and nuclease-free water to a final volume of 20 μl. The reaction conditions were as follows: 25°C for 5 min followed by 43°C for 60 min and 70°C for 15 min. The reverse transcription product was diluted 10‐fold and used as the template for quantitative real‐time PCR (Bio-Rad, Munich, Germany) and at least three replicates were performed for each gene. Primers for qPCR were designed for the first and fifth exon regions of *SABP2* and *SAMT* genes, respectively (S3 Table in S1 File). The relative expression levels of the genes were calculated using ‘delta Ct’ (ΔCt) methods and evaluated in the Bio-Rad CFX Manager software. The *ubiquitin* (S4 Table in S1 File) and *actin* (accession number: FJ410442) as the housekeeping genes.

## Results

The *Betula* species in Table 1, previously confirmed through barcoding and phylogenetic analyses [29], were used for *SAMT* and *SABP2* candidate gene identification and a comparative analysis.

### Identification of *SAMT* and *SABP2* candidate genes

The birch *SAMT* and *SABP2* candidate genes revealing the highest level of sequence similarities in *C*. *breweri (SAMT)* and *N*. *tabacum (SABP2)* were chosen for the phylogenetic analyses. For *SAMT*, three hits with similar E-values appeared after a tBlastn search [Bpev01.c0161.g0056.m0001 (*BpSAMT2*; E-value: 4E-47), Bpev01.c0161.g0057.m0001 (*BpSAMT3;* E-value: 9E-46) and Bpev01.c0425.g0055 (*BpSAMT;* E-value: 1E-45)] (S1 Table in S1 File). The phylogenetic tree (S1 Fig in S1 File) clearly shows that Bpev01.c0425.g0055 (*BpSAMT*) clustered closest to the reference *SAMT* gene from *C*. *breweri*, and was thus selected as the candidate gene for the analyses in this study. The coverage percentage between *CbSAMT* and *BpSAMT* was 61.8%, with 40.1% identity in the tBlastn search. A similar strategy was followed to identify putative *B*. *pendula SABP2* candidate genes. The three hits [Bpev01.c0161.g0056.m0001 (*BpSABP2*; E-value: 7E-46) Bpev01.c0161.g0057.m0001 (*BpSABP2-2*; E-value: 6E-36) and Bpev01.c0425.g0055.m0001 (*BpSABP2-3*; E-value: 6E-35)] with the lowest E-values were selected to construct a phylogenetic tree (S1 Fig in S1 File). The BpSABP2 (Bpev01.c0015.g0219) protein clustered closest to the reference NtSABP2, showed a 98% coverage and a 64.7% identity in the tBlastn search (S2 Fig in S1 File).

The *BpSAMT* gene is localized on chromosome IX and contains five exons and four introns, while *BpSABP2* is located on chromosome V and carries three exons and two introns. Using the *B*. *pendula* reference genes, specific primers (S3 Table in S1 File) were designed for *SAMT* and *SABP2* to amplify homologous regions in other *Betula* species, *B*. *alleghaniensis* (Ba), *B*. *lenta* (Bl), *B*. *medwediewii* (Bm), *B*. *grossa* (Bg), *B*. *utilis* (Bu), *B*. *alnoides* (Bal) and *B*. *nana* (Bn).

### Comparative analysis of SAMT and SABP2 protein sequences

Functionally characterized SAMT and SABP2 reference protein sequences from different species that showed enzymatic activity toward SA and MeSA, respectively, were compared with *B*. *pendula* SAMT and SABP2 proteins (S5 Table in S1 File) using the BLASTp option in the NCBI gene database.

BpSAMT displayed 55% coverage and 63.7% identity to the first functionally characterized CbSAMT (AF133053), and 50% coverage and 39.9% identity to AtBSMT1 (AT3G11480). A SABATH gene from *P*. *trichocarpa*, *PtSABATH4*, showed the highest activity towards SA, displaying 51% coverage and 54.37% identity to BpSAMT. BpSAMT is 95.5% (100%), 99.6% (100%), 96.9% (100%), 97.5% (100%), 93.5% (100%), 95.9% (100%) and 97.53% (99%) identical (coverage) to BaSAMT, BalSAMT, BlSAMT, BmSAMT, BuSAMT, BnSAMT and BgSAMT protein sequences, respectively.

Likewise, a comparative analysis of the BpSABP2 protein sequence with previously characterized genes from NtSABP2 showed 98% coverage (64.73% identity), while the *P*. *trichocarpa* PtSABP2-1 (Potri.007G037700) and PtSABP2-1 (Eugene3.00070971) showed 9% and 4% coverage and 28.6% and 40.9% identity, respectively, to BpSABP2. The BpSABP2 showed 53.5% identity and 96% coverage to the AtMES9 protein sequence that showed the highest enzymatic activity to MeSA in *A*. *thaliana*. BpSABP2 is 93.2% (100%), 96.2% (100%), 95.1% (100%), 98.1% (100%), 94.7% (100%), 97.2% (100%) and 93.5% (100%) identical (coverage) to BaSABP2, BalSABP2, BlSABP2, BmSABP2, BuSABP2, BnSABP2 and BgSABP2 protein sequences, respectively.

### Gene structure, sequence variation analysis and marker development

**Fig 1:**
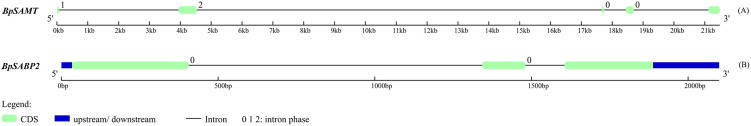
Structural features of *B*. *pendula SAMT* and *SABP2* genes. Exons are represented by round-corner rectangles, while the line between two exons represents an intron. Intron phases are represented by the numbers above the line. The intron phases are likely to assist in exon shuffling, recombination fusion, and protein domain exchange [45, 46].

The structure of *SAMT* and *SABP2* genes in the different birch species was predicted on the basis of the exon/intron organization of the homologous *B*. *pendula* genes (Fig 1). Based on these predictions, exon regions were amplified from four high (*B*. *alleganienisis* (*al*), *B*. *lenta* (*len*), *B*. *grossa* (*bg*) *B*. *medwediewii* (*med*)) and four low (*B*. *pendula* (*pen*), *B*. *utilis* (*util*), *B*. *nana* (nan) *B*. *alnoides* (*aln*)) MeSA-producing *Betula* species using various primer combinations to determine the nucleotide architecture of the *SABP2* and *SAMT* coding sequences (S3 Table in S1 File). Low sequence length variation in coding regions was observed within the eight *Betula* species (S4 Table in S1 File), ranging from 1,344 bp to 1,348 bp for *SAMT* and as 792 bp for *SABP2*.

A comparative analysis among the eight birch species displayed a considerable amount of nucleotide polymorphism within the genes. Here, putative group-specific single nucleotide polymorphisms (SNPs) were considered for marker analysis (Table 2). Groups were allocated on the basis of their ability to produce high and low levels of MeSA. In total, 38 individuals from different *Betula* species originating from different botanical gardens (S6 Table in S1 File) were analyzed for *SAMT* and *SABP2* gene sequence variation by designing specific primers (S3 Table in S1 File).

**Table 2:** Nucleotide characteristics and validation of SNPs discovered in *SABP2* gene regions to determine the eight high and low MeSA-producing *Betula* species. High MeSA (yellow): *B*. *alleganienisis* (ale), *B*. *lenta* (len), *B*. *grossa* (bg), *B*. *medwediewii* (med); low MeSA: *B*. *pendula* (pen), *B*. *utilis* (uti), *B*. *nana* (nan), *B*. *alnoides* (aln). Group-specific SNPs are shown in blue.

Nuclear region	Total number of SNP	Position	ale (6*)	len (6*)	bg (4*)	med (4*)	pen (6*)	uti (4*)	nan (4*)	aln (4*)
SABP2	6	160bp	S (6)	C (4)/S (2)	S (4)	S (4)	G (6)	G (4)	G (4)	G (4)
		189bp	R (6)	A (5) / R (1)	R (4)	R (3) / G (1)	G (6)	G (4)	G (4)	G (4)
		262bp	T (6)	T (6)	T (4)	T (2), W (1), A (1)	A (6)	A (4)	A (4)	A (4)
		298bp	G (6)	G (6)	G (3) / A (1)	G (2) / R (1) / A (1)	A (6)	A (4)	A (4)	A (4)
		304bp	G (5) / K (1)	K (6)	G (4)	K (3) / T (1)	T (6)	T (4)	T (4)	T (4)
		336bp	A (6)	A (6)	A (4)	R (4)	G (6)	G (4)	G (4)	G (4)

*Total number of species used to validate the discovered polymorphic sites.

In total, six prominent group-specific SNPs were discovered to differentiate between high (highlighted in yellow) and low MeSA-producing birch species (Table 2). The occurrence of heterozygous nucleotide sites was frequently observed in all high MeSA-producing species, while no heterozygous positions were observed in low MeSA-producing birches. Four of the six SNP positions revealed heterozygous sites within the group of high MeSA producers, with exception of *B*. *medwediewii* (med). In the latter species, all six SNP positions revealed heterozygous sites.

High nucleotide variations were also detected for the *SAMT* gene, however, no group-specific nucleotide substitution that is putatively associated with high or low MeSA-producing ability was observed.

### Functional domain and conserved motif analysis

**Fig 2:**
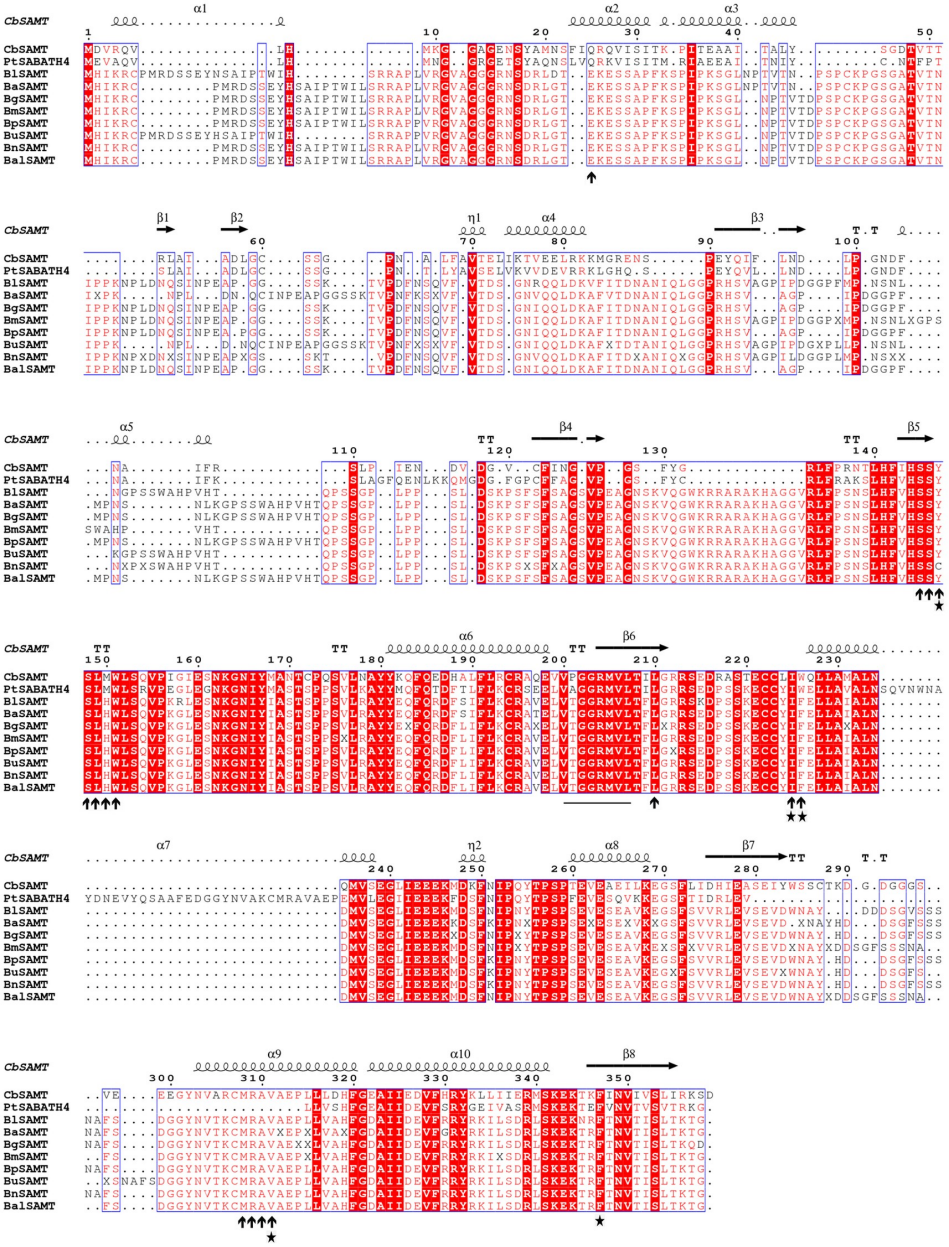
Multiple sequence alignment of SAMT amino acid sequences constructed from CbSAMT, *C*. *breweri* SAMT [4], PtSABATH4, *P*. *trichocarpa* SABATH [50] and eight *Betula* species, including *B*. *lenta* (BlSAMT), *B*. *alleghaniensis* (BaSAMT), *B*. *grossa* (BgSAMT), *B*. *medwediewii* (BmSAMT), *B*. *pendula* (BpSAMT), *B*. *utilis* (BuSAMT), *B*. *nana* (BnSAMT) and *B*. *alnoides* (BalSAMT). The blue frames represent the conserved residues, the white characters in red boxes suggest strict identity and the red characters in white boxes specify similarity. All the amino acid sequences carry the conserved domain of methyltransfer including the SAM-binding motif that had been previously defined, highlighted with green line [47]. The positions of residues involved in the SA substrate binding, identified from the three-dimensional structure [48], are indicated by arrows, while the residues indicated with a star are the aromatic moiety of the substrate and important for substrate selectivity [49]. The figure was prepared with ESPript [51].

The functional domain of the *Betula* SAMT amino acid sequences were analyzed and compared with the respective reference protein sequences. The Pfam (https://www.genome.jp/) domain search revealed that the methyltransferase 7 domain was conserved in all the SAMT protein sequences included in the study. All *Betula* SAMT only displayed domains described as SAM-dependent carboxyl methyltransferase (S7 Table in S1 File). Multiple sequence alignment of SAMT amino acid sequences was constructed using *Betula* and the respective reference amino acid sequences for structural analysis. The alignment of *B*. *lenta* (BlSAMT), *B*. *alleghaniensis* (BaSAMT), *B*. *grossa* (BgSAMT), *B*. *medwediewii* (BmSAMT), *B*. *pendula* (BpSAMT), *B*. *utilis* (BuSAMT), *B*. *nana* (BnSAMT), *B*. *alnoides* (BalSAMT) together with CbSAMT shown to contain the conserved domain of methyltransferase (Fig 2). In addition, we detected the occurrence of a previously defined [47] SAM-binding motif within the aligned sequences. Further, we observed the positions of residues involved in the substrate-binding site that was identified from the three dimensional structure [48] and the aromatic moiety of the substrate that is important for substrate selectivity of SAMT proteins [49] (Fig 2).

**Fig 3:**
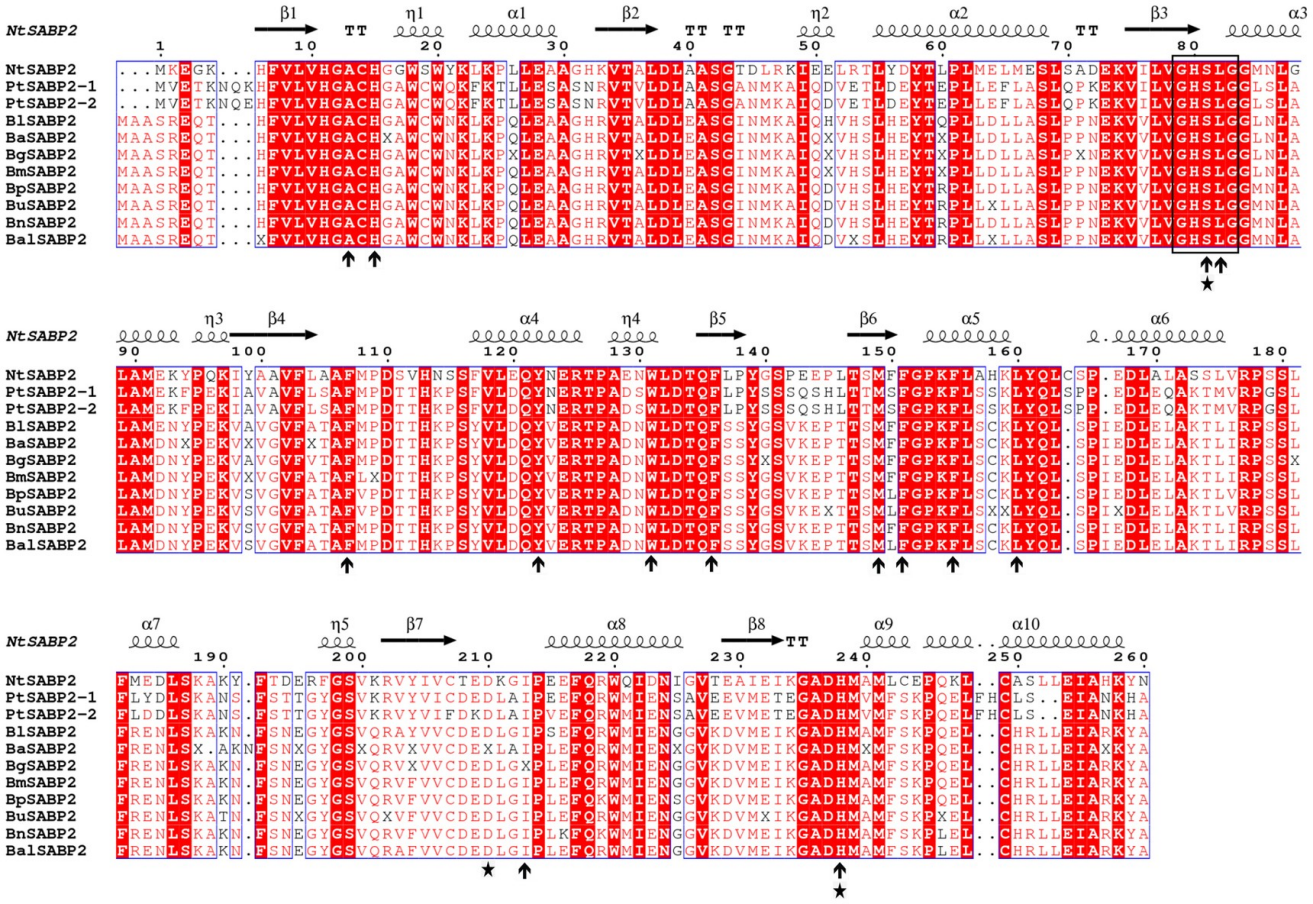
Multiple sequence alignment of SABP2 amino acid sequences constructed from *N*. *tobacco* SABP2 (*NtSABP2*) [7], *P*. *trichocarpa* SABP2 (*PtSABP2-1*, *PtSABP2-2*) [53] and eight *Betula* species, including *B*. *lenta* (BlSAMT), *B*. *alleghaniensis* (BaSAMT), *B*. *grossa* (BgSAMT), *B*. *medwediewii* (BmSAMT), *B*. *pendula* (BpSAMT), *B*. *utilis* (BuSAMT), *B*. *nana* (BnSAMT) and *B*. *alnoides* (BalSAMT). The blue frames represent the conserved residues, the white characters in red boxes suggest strict identity and the red characters in white boxes specify similarity. The lipase signature sequence of SABP2 is displayed with black frame. The three conserved amino acids forming a catalytic triad, S81, D210 and H238, commonly found in the hydrolase domain, are indicated with a star and are conserved in *Betula* SABP2 [7], while residues that contact to SA are indicated by arrows [5]. The figure was prepared with ESPript [51].

Similarly, another multiple amino acid sequence alignment (Fig 3), including *B*. *lenta* (BlSABP2), *B*. *alleghaniensis* (BaSABP2), *B*. *grossa* (BgSABP2), *B*. *medwediewii* (BmSABP2), *B*. *pendula* (BpSABP2), *B*. *utilis* (BuSABP2), *B*. *nana* (BnSABP2), *B*. *alnoides* (BalSABP2) and the reference NtSABP2, was constructed. The α/β hydrolase-6 domain was conserved in all tested SABP2 protein sequences. The *Betula* SABP2 protein sequences displayed multiple domains, including α/β hydrolase*-*1, α/β hydrolase*-*4 and Lipase-3. We observed the three conserved amino acids form the catalytic triad that is commonly found in the hydrolase domain and the residues that contact SA [52] (Fig 3).

The MEME online tool was used to identify the conserved motifs and/or differences in protein structure among the *Betula* SAMT and SABP2 amino acid sequences. In total, 11 and five equally shared conserved motifs were observed in all the *Betula* SAMT and SABP2 amino acid sequences, respectively (S3A and S3B Fig in S1 File).

### Phylogenetic analysis and functional prediction

To ascertain the evolutionary relationship of the *Betula* SAMT and SABP2 with the SAMT and SABP2 members of other plant species which have been functionally characterized (S2 Table in S1 File), a maximum likelihood phylogenetic tree with 1,000 bootstrap values was constructed using the amino acid sequences of *B*. *lenta* (BlSAMT and BlSABP2), *B*. *alleghaniensis* (BaSAMT and BaSABP2), *B*. *grossa* (BgSAMT and BgSABP2), *B*. *medwediewii* (BmSAMT and BmSABP2), *B*. *pendula* (BpSAMT and BpSABP2), *B*. *utilis* (BuSAMT and BuSABP2), *B*. *nana* (BnSAMT and BnSABP2) and *B*. *alnoides* (BalSAMT and BalSABP2). The possible substrate specificity of *Betula* SAMT and SABP2 proteins was determined on the basis of the phylogenetic clustering genes in the same subgroup, and might share a similar function.

**Fig 4:**
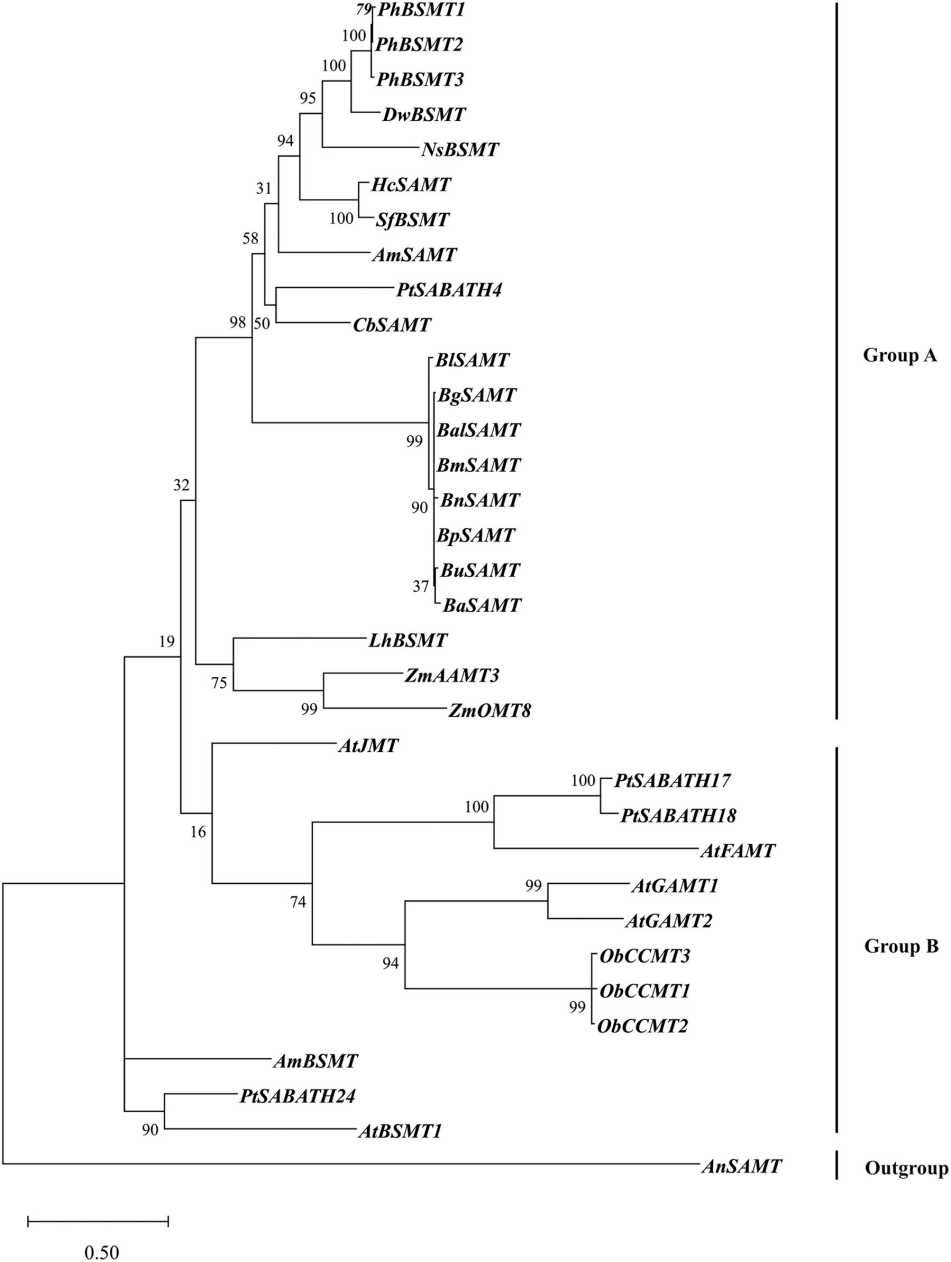
Evolutionary relationship of *Betula* SAMT proteins: The phylogenetic tree was constructed using amino acid sequences of *B*. *lenta* (Bl SAMT), *B*. *alleghaniensis* (Ba SAMT), *B*. *grossa* (Bg SAMT), *B*. *medwediewii* (Bm SAMT), *B*. *pendula* (Bp SAMT), *B*. *utilis* (Bu SAMT), *B*. *nana* (Bn SAMT) and *B*. *alnoides* (Bal SAMT) species with 26 functionally characterized SAMT from other species (S2 Table in S1 File). A total number of 34 SAMT amino acid sequences were used in the maximum likelihood method in the MEGA7 software [43]. A SAMT from *Aspergillus niger* (NT166520) was used as an outgroup species. The numbers at the nodes indicate bootstrap values calculated with 1,000 replicates. Branches are drawn to scale with the bar indicating 0.50 substitutions per site.

According to the phylogenetic tree (Fig 4), the SAMT proteins were divided into two groups (Group A and B). All the *Betula* SAMT was clustered together in Group A with the SAMT from *P*. *trichocarpa* (PtSABATH4) and *C*. *breweri* (CbSAMT). In addition, the *Betula* SAMT was clustered in a subgroup of Group A having bootstrap values of 98 and accompanying the SAMT from other species, suggesting that *Betula* SAMT most probably shares a similar function. The SAMT with a higher homology infers the function of the unknown *Betula* SAMT according to the clustering of the phylogenetic tree. These SAMT proteins all use SA as a substrate that synthesizes the volatile ester MeSA. It should be noted that *Arabidopsis* BSMT (AT3G11480), which uses both SA and benzoic acid (BA) as a substrate, is not clustered together with *Betula* SAMT.

**Fig 5:**
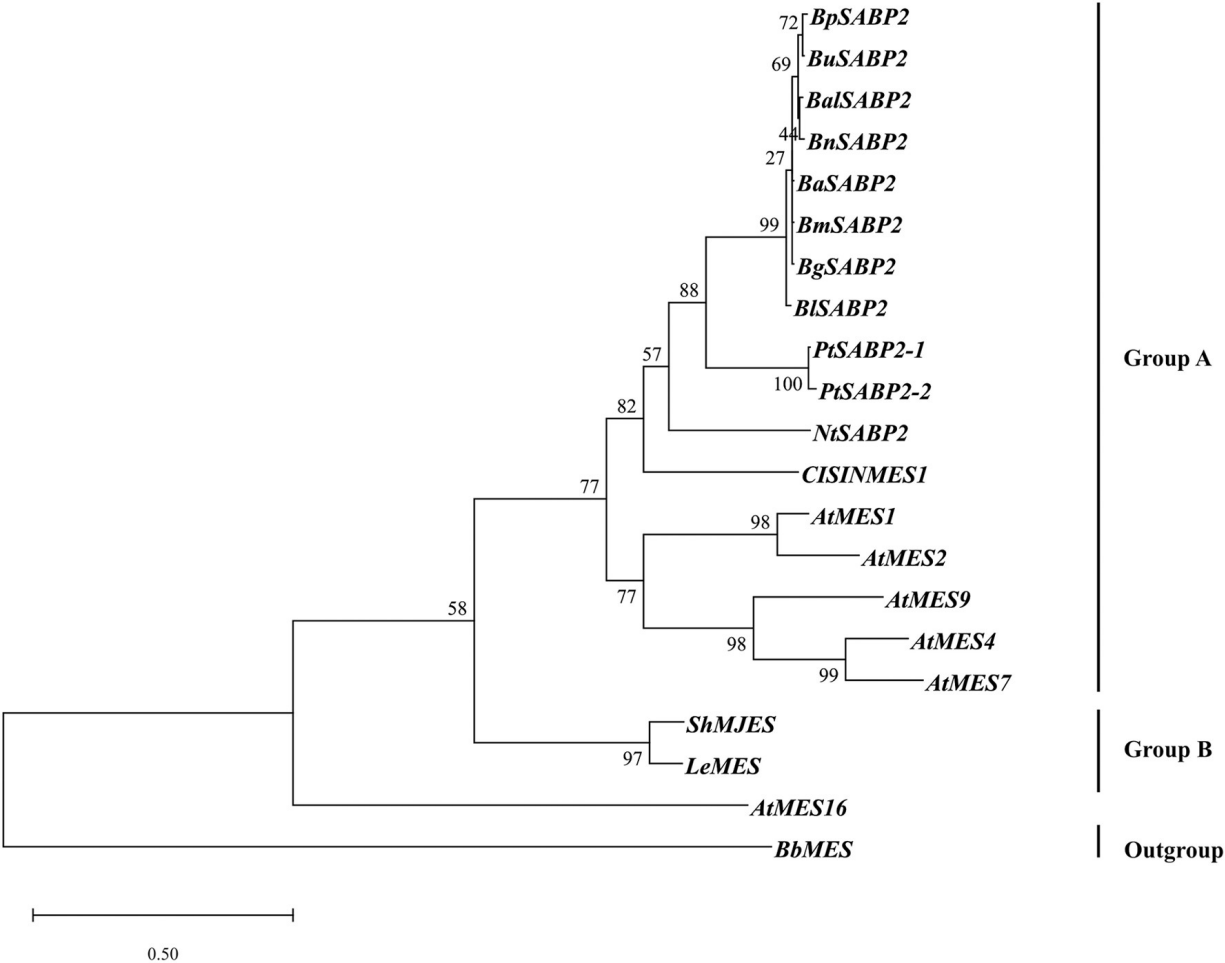
Evolutionary relationship of *Betula* SABP2 proteins: The phylogenetic tree was constructed using amino acid sequences of *B*. *lenta* (BlSABP2), *B*. *alleghaniensis* (Ba SABP2), *B*. *grossa* (Bg SABP2), *B*. *medwediewii* (Bm SABP2), *B*. *pendula* (Bp SABP2), *B*. *utilis* (Bu SABP2), *B*. *nana* (Bn SABP2) and *B*. *alnoides* (Bal SABP2) species with 13 functionally characterized SABP2/MES from other species (S2 Table in S1 File). A total number of 21 SABP2/MES amino acid sequences were used in the maximum likelihood method in the MEGA7 software [43]. A MES from *Beauveria bassiana* (PMB68924.1) was used as an outgroup species. The numbers at the nodes indicate bootstrap values calculated with 1,000 replicates. Branches are drawn to scale with the bar indicating 0.50 substitutions per site.

Similarly, the SABP2 phylogenetic tree (Fig 5) was constructed using the *Betula* SABP2 protein with other known SABP2 proteins from different plant species (S2 Table in S1 File). *Betula* SABP2 clustered in Group A together with the functionally characterized SABP2 from *P*. *trichocarpa* (PtSABP2-1 and PtSABP2-1) with a bootstrap value 88 for the clade, suggesting a possible functional similarity. The *Arabidopsis* MESs (AtMES1, 2, 4, 7 and 9) and NtSABP2 also clustered in Group A.

The candidate and reference proteins formed a clade in the phylogenetic tree (Figs 4 and 5) and were also included in the sequence alignment analysis (Figs 2 and 3).

The intraspecific evolutionary relationship of *SAMT* and *SABP2* in eight high and low MeSA-producing *Betula* species were also analyzed by constructing two phylogenetic trees using the maximum likelihood method in MEGA X software [43]. The exon regions of the *SAMT* and *SABP2* genes were sequenced for all the *Betula* species and converted into the amino acid sequences. Both the phylogenetic trees revealed two clades differentiating the high and low MeSA-producing birch species (S4A and S4B Fig in S1 File).

### Expression analysis of *SAMT* and *SABP2* in different birch species and tissues

**Fig 6:**
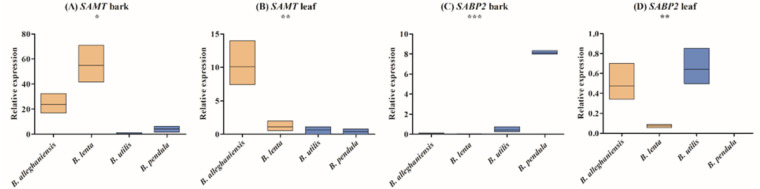
Tissue-specific expression of the *SAMT* and *SABP2* genes in two high (*B*. *alleghaniensis* and *B*. *lenta*; gray-tan columns) and two low (*B*. *utilis* and *B*. *pendula*; blue columns) MeSA-producing birch species. The expression of candidate genes was assessed by qRT-PCR. The y-axis indicates the relative expression level, while the x-axis indicates the different tissue of the different species. Three biological and three technical replicates were used. *Actin* and *ubiquitin* reference genes were used to normalize the expression.

To detect the prior tissue-specific expression of *SAMT* and *SABP2* genes in *Betula*, we analyzed the expression of *SAMT* and *SABP2* in the leaf and bark of two high MeSA (*B*. *lenta* and *B*. *alleghaniensis*) and two low MeSA (*B*. *utilis* and *B*. *pendula*) producers using quantitative real-time RT-PCR (Fig 6). Altogether, *SAMT* and *SABP2* genes revealed differential expression patterns in the two tissues analyzed from high and low MeSA producers. The *B*. *alleghaniensis SAMT* (*BaSAMT*) displayed high expression in the bark as well as in the leaf (Fig 6A and 6B) tissues, while *B*. *lenta SAMT* (*BlSAMT*) was highly expressed only in the bark (Fig 6A and 6B). Both *B*. *utilis SAMT* (*BuSAMT*) and *B*. *pendula SAMT* (*BpSAMT*) had a low expression in both bark and leaf tissue.

For *SABP2*, *B*. *alleghaniensis* (*BaSABP2*) and *B*. *lenta* (*BpSABP2*) showed no expression in the bark and low expression in leaves (Fig 6C and 6D). On the other hand, *B*. *utilis SABP2* (*BuSABP2*) showed a low expression in the bark and a high expression in the leaf, while *B*. *pendula SABP2* (*BpSABP2*) was highly expressed only in the bark.

### *Betula SAMT* and *SABP2* gene promoter analysis

The sequences obtained from the PCR-amplified promoter regions of the *SABP2* and *SAMT* genes from the low MeSA-producing *B*. *pendula* (pen), *B*. *utilis* (uti), *B*. *nana* (nan), *B*. *alnoides* (aln), and the high MeSA-producing *B*. *alleganienisis* (ale), *B*. *lenta* (len), *B*. *grossa* (bg), *B*. *medwediewii* (med) were submitted to the NCBI database (S8 Table in S1 File). All sequences were analyzed using PlantCARE [44] to identify putative *cis* elements (S9 and S10 Tables in S1 File). We obtained *BpSAMT* and *BpSABP2* gene promoter regions from the *B*. *pendula* genome sequence [42]. The length of the *BpSABP2* and *BpSAMT* promoters were 1,050 bp and 1,003 bp, respectively. The results indicated that the promoter regions contain multiple eukaryotic *cis*-acting elements, including TATA and CAAT boxes. In the *BpSABP2* promoter sequence, four abscisic acid response elements (ABRE) were found at positions bp 74+, 191-, 938- and 939+; three Box4 parts of conserved DNA module elements were located at positions bp 143+, 801- and 581-; one Sp1 at position bp863- and three G-Box light-responsive elements were located at positions bp 73-, 983- and 191+. Two light-responsive GATA-motifs were localized at positions bp 454- and 766+; two elements involved in circadian control were located at positions bp 964+ and 973+ and one auxin-responsive element (TGA-element) was found at position bp 62+. The *BpSAMT* contained the plant light-responsive elements (GTGGC-motifs) at position bp 170; a chs-CMA2a at position bp 75- and 246-, and one auxin responsive AuxRR-core element was located at position bp 714-.

**Fig 7:**
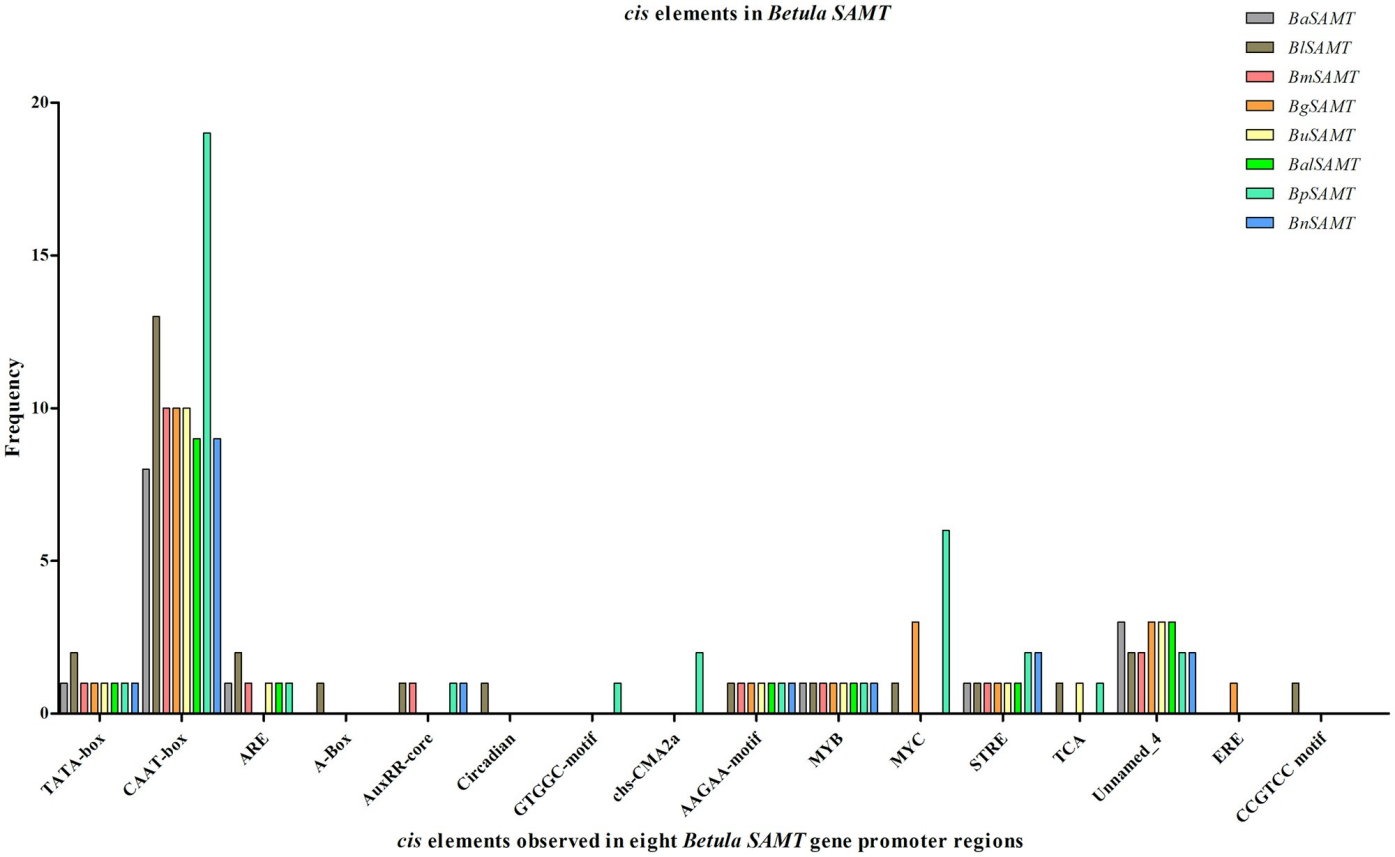
Frequencies of identified *cis* elements using the PlantCARE database [44] in the promoter regions of *SAMT* genes of four high (*B*. *lenta* (Bl), *B*. *alleghaniensis* (Ba), *B*. *grossa* (Bg) and *B*. *medwediewii* (Bm)) and four low (*B*. *pendula* (Bp), *B*. *utilis* (Bu), *B*. *nana* (Bn) and *B*. *alnoides* (Bal)) MeSA-producing birch species.

**Fig 8:**
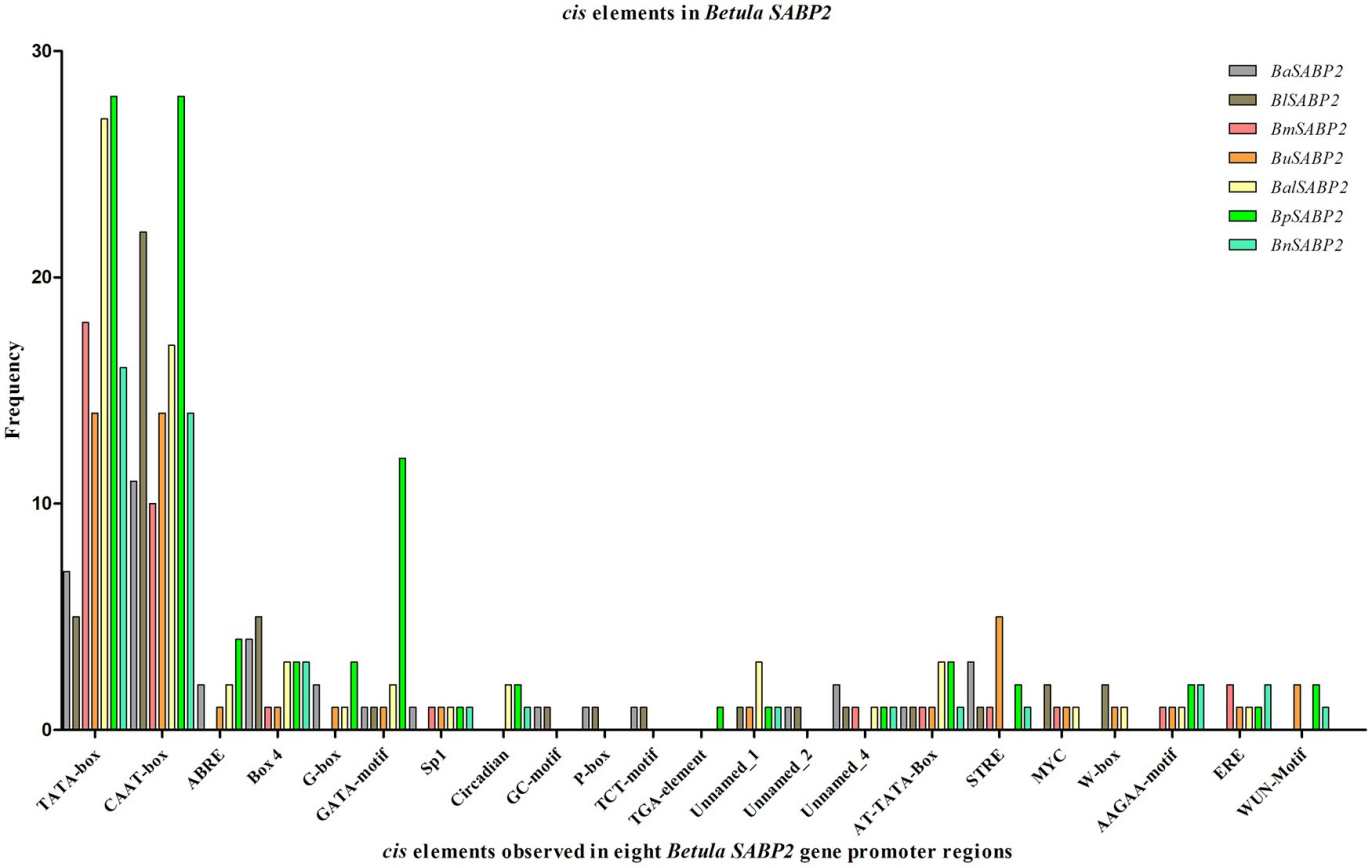
Frequencies of identified *cis* elements using the PlantCARE database [44] in the promoter regions of *SABP2* genes of three high (*B*. *lenta* (Bl), *B*. *alleghaniensis* (Ba) and *B*. *medwediewii* (Bm)) and four low (*B*. *pendula* (Bp), *B*. *utilis* (Bu), *B*. *nana* (Bn) and *B*. *alnoides* (Bal)) MeSA-producing birch species. Unfortunately, the promoter region of *B*. *grossa* (Bg) could not be amplified.

Through the primer walking approach, about 600 bp and 700 bp of the *SAMT* and *SABP2* promoter regions were also successfully obtained for the eight other *Betula* species (except for *B*. *grossa SABP2*) for comparative analysis (S8 Table in S1 File). The presence of different *cis* elements, together with their frequencies in the *SAMT* and *SABP2* gene promoter regions, was evaluated in seven birch species (Figs 7 and 8, respectively). The fragment length of *SAMT* and *SABP2* promoters varied between 603–628 and 636–770 base pairs, respectively (S9 and S10 Tables in S1 File). A comparative analysis of the *cis* regulatory elements revealed considerable differences in the frequencies between the high and low MeSA producers *B*. *lenta* and *B*. *pendula*, respectively. The *B*. *lenta SAMT* promoter region showed two TATA boxes, while all other species contains only one TATA box. Different numbers of TATA boxes were observed in the *SABP2* gene promoter regions of *B*. *pendula* and *B*. *lenta*.

## Discussion

Intensive studies have been conducted to reveal the role of MeSA in plant immunity and the signaling cascades of the SAR mechanism in plants [3, 54, 55]. SAMT, which belongs to the SAM-dependent methyltransferases and is commonly found in plants, forms MeSA by the methylation of SA [3]. MeSA reverts to SA by SABP2 under strong esterase activity [2, 6]. Almost all plants produce MeSA as a long-distance mobile signal in stress defense and as an SAR mechanism [1], including the birch species from the subgenus *Betula* (*B*. *utilis*, *B*. *pendula*, *B*. *nana* and *B*. *alnoides*), denoted “low MeSA producers” [29]. However, in addition to its role in immunity and the SAR signaling cascade, MeSA is produced constitutively in some winter green shrubs and birch species, and is therefore believed to be an important plant constituent [29, 30]. Constitutive MeSA production is expressed by a natural sweet and strong scent and has medicinal and pharmaceutical significance [31–33]. *Betula* species that constitutively produce MeSA are called “high MeSA producers.” They belong to the subgenus *Aspera* and include *B*. *alleghaniensis*, *B*. *lenta*, *B*. *grossa* and *B*. *medwediewii* [29, 30].

It has been confirmed that SAMT and SABP2 enzymes mediate MeSA biosynthesis in many plant species [19, 52–54, 56, 57]. In particular, both enzymes have been functionally characterized and well-studied in different plant species, including *N*. *tabacum*, *C*. *breweri*, *P*. *trichocarpa*, *V*. *vinifera*, and *A*. *thaliana* [4, 50, 52, 54]. However, very little is known about the genetic architecture of SAMT and SABP2 in the ecologically important tree species of the genus *Betula*.

In this study, we identified two candidate genes, *SAMT* and *SABP2*, in low and high MeSA-producing birch species using protein sequences of previously functionally characterized from *C*. *breweri* SAMT [4] and *N*. *tabacum* SABP2 [7] as references. Also, to the best of our knowledge, this is the first study that has identified putative high and low MeSA-specific nucleotides in the *SABP2* gene that could be used to develop molecular markers to differentiate high and low MeSA-producing *Betula* species.

### Marker development and validation

Although hybridization, introgression and misidentification have often been obstacles in the systematics of the genus *Betula* [58, 59], the characteristics of leaf shape, bark color, and varying chemical composition of the bark and leaves have successfully been applied to classify the majority of birch species [36–38]. Until now, the classification of some birch species is still under discussion, with several taxonomical classifications still in existence for the genus *Betula* [30, 58, 60, 61]. The use of molecular markers based on, e.g. microsatellites (SSR) and chloroplast regions, could help resolve systematics issues and this technique has already been widely introduced into plant systematics [62–64]. Next-generation sequencing technologies have recently made it possible to conduct whole genome sequencing, allowing the generation of a large number of genome-wide markers [65–67]. Population genetics studies of the silver birch have identified genetic variations in genes that are associated with local adaptations to different environmental conditions [42].

For the genus *Betula*, low and high MeSA-synthesis ability could be an additional criterion for species systematics in this genus [29, 30]. Therefore, we defined the *SAMT* and *SABP2* candidate genes involved in the MeSA biosynthesis of eight *Betula* species, including *B*. *lenta*, *B*. *alleghaniensis*, *B grossa*, *B*. *medwediewii* (high MeSA-producing), *B*. *utilis*, *B*. *pendula*, *B*. *alnoides* and *B*. *nana* (low MeSA-producing).

Various studies have advocated the importance of *SABP2* and *SAMT* genes in plant developmental stages and signaling cascades [3, 7, 50, 52, 68]. Considering their interaction with numerous molecules, *SABP2* and *SAMT* displayed a higher percentage of polymorphism compared to previously analyzed genomic regions in *Betula* [64, 69, 70]. The relative analysis is in agreement with our earlier study where small segments of both genes were investigated [29].

Although significant nucleotide variation was observed between all *Betula* species analyzed, only low and high MeSA-specific nucleotides were considered. We discovered six specific positions on the *SABP2* gene that could be associated with high MeSA production in birches (Table 2). Validation of the nucleotide substitutions (SNPs) was performed with 38 additional birch individuals belonging to eight different birch species from different botanical gardens (S6 Table in S1 File), indicating that species-specific nucleotide substitutions are associated with high MeSA production. To the best of our knowledge, this is the first study that has attempted to identify high and low MeSA-specific nucleotides in the *SABP2* gene in different *Betula* species that could be used to develop SNP markers associated with low and high MeSA content. Unfortunately, in the *SAMT* gene, no high or low MeSA-specific nucleotides could be detected. However, the SNPs identified in the *SABP2* gene need to be validated in additional low and high MeSA-producing birch species and by including more individuals.

The decaploid *B*. *medwediewii* (subgenus, *Aspera*; section, *Lentae*) exhibited considerable heterozygous nucleotides at all six SNP positions in the *SABP2* gene (Table 2). Ashburner et. al., (2013) revealed *B*. *medwediewii* as a high MeSA producer, while the olfactory fragrance analysis unanimously categorized *B*. *medwediewii* as an intermediate MeSA producer [29]. In addition to the presence of substantial heterozygous SNPs in the *SABP2* gene and intermediate MeSA production, the clustering of *B*. *medwediewii* with the species of the subgenus *Betula* [29] supports the idea that during the evolution of this species, one of the parents belonged to the subgenus *Betula*. Its partial MeSA-producing ability could be a rational motivation for Ashburner et al., (2013) allocating this species to the subgenus *Aspera*.

### Comprehensive bioinformatics analysis

The genetic architecture of the silver birch has been recently enhanced due to the available genome. In our study, we used different bioinformatics tools, including sequencing, gene structure analysis, multiple sequence alignment, domain characterization, conserved motifs, promoter analysis and phylogenetic relationships analysis. The aim was to collect vital information on the different high and low MeSA-producing birch species for biotechnological purposes, including functional analysis, molecular breeding and the commercial use of natural medicinal products.

All the *Betula SAMT* candidate genes from eight different birch species in the study showed the presence of a methyltransferase 7 domain (Methyltransf_7; S7 Table in S1 File) and a conserved motif III that possess SAM-binding sites described previously (Joshi et al., 1998). The occurrence of the motif III in 56 different plant species suggests it plays a major role in the binding of the SAM-dependent *O-*methyltransferases to their specific substrate, which also includes SAMT that catalyzes SA into MeSA [47, 71]. The crystallography analysis of the *C*. *breweri* SAMT protein and the substrate SA complex possesses active sites responsible for the selection of SA that were also characterized in *Betula* SAMT [48], suggesting its role in MeSA biosynthesis (Fig 2).

Amino acid sequences of *Betula* and *C*. *breweri* SAMT proteins revealed only three mismatches: the *Betula* SAMT has histidine, phenylalanine and tyrosine at positions 150, 209 and 224, rather than methionine, isoleucine and leucine, respectively, in the *C*. *breweri* SAMT [48] (Fig 2). In total, 14 SA binding residues were identified in the *Betula* SAMT, compared to 16 in *C*. *breweri* [48].

The alignment of the *Betula* SAMT (Fig 2) and previously functionally characterized members of SABATH family suggests that *Betula* SAMT probably methylates both SA and the structurally similar substrate BA [54]. It has also been experimentally proven that members of the SABATH methyltransferase family catalyze multiple substrates with different *K*_*m*_ values [8, 50, 54, 55]. Additionally, it has been suggested that a single amino acid substitution might play a critical role in the specificity of SAMT/BSMT with SA and BA [72]. Further, the detailed study by Han et al., (2017) on *P*. *trichocarpa* revealed the evolutionary substrate specificity of the members of the methyltransferase family, including SAMT, can be achieved by changes in amino acid sequences and that alterations in a single amino acid might result in a divergence in substrate specificity [50]. Despite the cited study, the actual mechanism behind the substrate specificity of the SAMT enzyme is still unclear. However, through structural analyses, it has been suggested that the size and shape of the active sites may play an important role in the differentiation of individual substrates [73]. The *Betula* SAMT protein alignment also revealed the presence of hydrophobic and aromatic-rich residues of the carboxyl bearing substrate-binding pockets that were previously observed in the detailed study of *A*. *thaliana* indole-3-acetic acid methyltransferase (AtIAMT) and CbSAMT [4, 49] (Fig 2).

Likewise, the amino acid sequence alignment of SABP2 from eight different birch species revealed the presence of a catalytic α/β hydrolase domain (Abhydrolase_6; S7 Table in S1 File) conserved in the SABP2 family which is in agreement with *A*. *thaliana* SABP2/MES enzymes [74]. The signature amino acid sequence, conserved in the *N*. *tobacco* SABP2 [5], was recognized in all eight *Betula* SABP2 species (Fig 3). All eight *Betula* SABP2 displayed the conserved catalytic triad found in the hydrolase domain that was proved in the protein profiling of *N*. *tobacco* SABP2 [7]. The conserved catalytic triad is in agreement with previous analyses conducted with *Arabidopsis* and the grapevine [52, 74]. Moreover, the 14 residues observed in *Betula* SABP2 that contact to SA were consistent with a previous structural study of tobacco SABP2 [5].

The phylogenetic tree revealed that SAMT from the investigated *Betula* species cluster together with the first functionally characterized *C*. *breweri* SAMT [4] and *P*. *trichocarpa* PtSABATH4 (Fig 4). It is noteworthy that the eight *Betula* and *Populus* SAMT (PtSABATH4) sequences clustered more closely to known SAMTs from *Antirrhinum majus* and *Hoya carnosa* flowers. Additionally, *Stephanotis floribunda*, *Nicotiana suaveolens*, *Datura wrightii* and *Petunia hybrid* BSMT (benzoic acid/salicylic acid methyltransferase) were also clustered in the same clade. The clustering of SAMT and BSMT might have occurred since the purified SAMT enzymes from *C*. *breweri*, and *S*. *floribunda* are able to methylate both SA and BA with higher and lower affinity, respectively [4, 75, 76]. In addition to the SAMT enzymes in Group A, the methyltransferases with different substrate specificity clustered in a paraphyletic Group B containing, for example, *Arabidopsis* jasmonic acid carboxyl methyltransferase (AtJMT) (Fig 4). It was hypothesized that JMT and SAMT/BSMT might have evolved from the indole-3-acetic acid carboxyl methyltransferase (IAMTs) [49].

The *SABP2* phylogeny, with functionally characterized genes from other species, showed *SABP2-1* and *SABP2-2* from *P*. *trichocarpa* clustered together with 94 bootstrap values (Fig 5). The two copies of the *SABP2* gene in *P*. *trichocarpa* were most probably the result of genome duplication events [77], while no signs of duplication events were observed in *B*. *pendula*, resulting in only one copy of *SABP2* in the investigated *Betula* species [42]. Both *PtSABP2-1* and *PtSABP2-2* genes showed explicit esterase activity to MeSA that produced salicylic acid [53]. Since the *Betula* species and *P*. *trichocarpa* are both woody plants and *Betula* SABP2 and PtSABP2 occur in one clade, we can predict that the *Betula* SABP2 functions similarly to PtSABP2. Although the *Betula* candidate genes showed low coverage and identity (S5 Table in S1 File) to the most closely related species, *P*. *trichocarpa*, still they formed a single clade. Therefore, we also recommend using phylogenetic analysis as a candidate gene selection criterion.

Additionally, NtSABP2, PtSABP2 and all eight *Betula* SABP2 displayed the three conserved amino acids forming a catalytic triad. We therefore hypothesize that *Betula* SABP2 catalyzes MeSA with its esterase activity (Fig 3). A comparative analysis of identity (coverage) of *Betula* SABP2 with functionally characterized Arabidopsis AtMES1, AtMES2, AtMES4, AtMES7 and AtMES9 protein sequences resulted in 58% (100%), 53% (100%), 54% (97%), 52% (97%), 54% (96%), respectively. All *Arabidopsis* AtMES1, 2, 4, 7 and 9 showed esterase activity towards SA [9] and the phylogenetic tree also suggests their evolutionary closeness with *Betula* SABP2 (Fig 5). It has been shown that AtMES proteins are responsible for the hydrolysis of other methyl esters, suggesting that almost of all these proteins are able to utilize multiple substrates with different enzymatic activity [74].

Studies of the eukaryotic promoter have shown that gene transcription activity is controlled by multiple cis and trans-acting elements [78]. Detailed studies of these elements were obtained from diverse experiments, including deletion, element relocation and mutagenesis analysis [79]. Considering the importance of cis elements, we successfully amplified the promoter regions of all the *Betula SAMT* and *SABP2* genes used for analyzing the functions of regulatory elements. We conducted a detailed comparative analysis between high (*B*. *alleghaniensis*, *B*. *lenta*, *B*. *grossa* and *B*. *medwedweii*) and low (*B*. *pendula*, *B*. *utilis*, *B*. *alnoides* and *B*. *nana*) MeSA producers. The *Betula SAMT* and *SABP2* gene promoters contain a variety of common elements, including the TATA and CAAT boxes (Figs 7 and 8). The promoter region of *B*. *lenta SAMT* showed two TATA boxes, while the *SAMT* of all the other *Betula* had only one. In the case of *SABP2* promoters, the low MeSA producers, *B*. *utilis*, *B*. *nana*, *B*. *alnoides* and *B*. *pendula*, displayed a higher number of TATA boxes compared to the high MeSA producers *B*. *lenta* and *B*. *alleghaniensis* (Figs 7 and 8). The occurrence of additional transcription starting sites suggests a higher likelihood of relevant expression, since *B*. *pendula* is a low MeSA-producing birch. The only functionally known *cis*-acting element involved in the circadian rhythm was observed in the promoter regions of both genes and the collective analysis indicated that both *SAMT* and *SABP2* might be induced by the plant hormones [5, 80, 81].

### Expression analysis

In order to detect the possible tissue-specific expression of *SAMT* and *SABP2* genes in high (*B*. *lenta* and *B*. *alleghaniensis*) and low (*B*. *pendula* and *B*. *utilis*) MeSA-producing *Betula* species, we analyzed the expression of *Betula SAMT* and *SABP2* genes in the bark and leaves of three-year-old plants.

The expression of *SAMT* was higher in the bark of *B*. *alleghaniensis* and *B*. *lenta* than in *B*. *pendula* and *B*. *utilis* (Fig 6A), suggesting its importance in the bark of high MeSA producers. The high expression of the *SAMT* gene in *B*. *alleghaniensis* and *B*. *lenta* species also reveals its significance in high MeSA production, since MeSA could be extracted in abundance from the stems of these plants [29, 31].

The characterized activity of *SAMT* in *C*. *breweri*, *S*. *floribunda* and snapdragons showed that the enzyme can methylate both SA and BA at different *K*_*m*_ values [4, 75, 76]. In *P*. *trichocarpa*, PtSABATH4 showed a higher enzymatic activity towards SA than BA, and a higher expression in all tissues studied when compared to other family members of SABATH [50]. Likewise, under normal growth conditions, the *A*. *thaliana BSMT1 (AtBSMT1)* and *A*. *lyrata BSMT1 (AlBSMT1)* genes showed considerable expression in leaves. In contrast, the AtBSMT1 protein showed higher enzymatic activity towards SA than BA, while the AlBSMT1 protein had a lower affinity for SA than BA [54]. The studies showed diversions within the substrate specificity of SA/BAMT proteins and collectively suggest that the *Betula SAMT* candidate gene could putatively also catalyze both SA and BA with divergent *K*_*m*_ values. This hypothesis also supports the deduced amino acid sequence of *Betula* SAMT, aligned with *C*. *breweri* and *Populus* SAMT (Fig 2), where amino acid shifts were observed. Considering the already published expression analysis of *O-methyltransferase* genes in poplar [82], *Arabidopsis* [83], citrus [84] and the results in this paper, we hypothesize that SAMT, which methylates SA to form MeSA, is highly expressed in the bark of the high MeSA producers *B*. *lenta* and *B*. *alleghaniensis*, resulting in the constitutive production of MeSA. Our hypothesis is in agreement with a previous SAMT analysis conducted in *C*. *breweri* flowers in order to characterize the molecules responsible for scent production [4].

For the first time, the SABP2 enzyme was identified in tobacco [85] and was shown to be a MeSA esterase and an important protein that is required for SAR development [5, 7]. In addition, the members of MES/SABP2 family have been isolated and characterized in many other plant species, including the grapevine [52], the potato [86], citrus [80] and poplars [53]. SABP2 is one of the many crucial elements of the SA signaling cascade that was identified by conducting intensive biochemical and molecular genetics studies in different plant species [53, 80, 87]. The bioinformatics sequence analysis of the *Arabidopsis* genome revealed 20 genes coding for proteins with relatively high sequence similarities to the tobacco *SABP2* [5, 74]. This suggests that methylesterases are involved in the hydrolysis of MeSA [74, 88].

The expression of *SABP2*, which converts MeSA into SA, was higher in the bark of the low MeSA-producing species *B*. *utilis* and *B*. *pendula* than in *B*. *alleghaniensis* and *B*. *lenta* (Fig 6C). The results suggest an abundance of SABP2 in *B*. *utilis* and *B*. *pendula*, and thus a higher affinity for reverting MeSA to SA. Likewise, in poplars, *SABP2-1* and *SABP2-2* showed the highest and a moderate level of expression in leaves and bark, respectively, while the expression of *PtSABP2-2* was found to be low in leaves under “normal” growing conditions [53].

To the best of our knowledge, this is the first study that has attempted to gather information about the genes involved in the biosynthesis of MeSA in birches. Detailed bioinformatics studies and expression analysis have led to the identification of candidate genes in eight species of the genus *Betula* that mediate MeSA biosynthesis. The results obtained in this study will be beneficial for further functional and enzymatic substrate specificity analysis of the *SAMT* and *SABP2* genes. In addition, this is the first attempt to identify high and low MeSA-specific nucleotides which can be used to develop SNP markers associated with low and high MeSA content for molecular breeding purposes.

## Supporting information

S1 File (DOCX)
