# Supplementary material for: Identification and analysis of key genes involved in methyl salicylate biosynthesis in different birch species
Source: PLoS One. 2020 Oct 8;15(10):e0240246. doi: 10.1371/journal.pone.0240246 (PMC7544025; doi:10.1371/journal.pone.0240246)
Supplement: S1 File — (DOCX) [file pone.0240246.s001.docx]

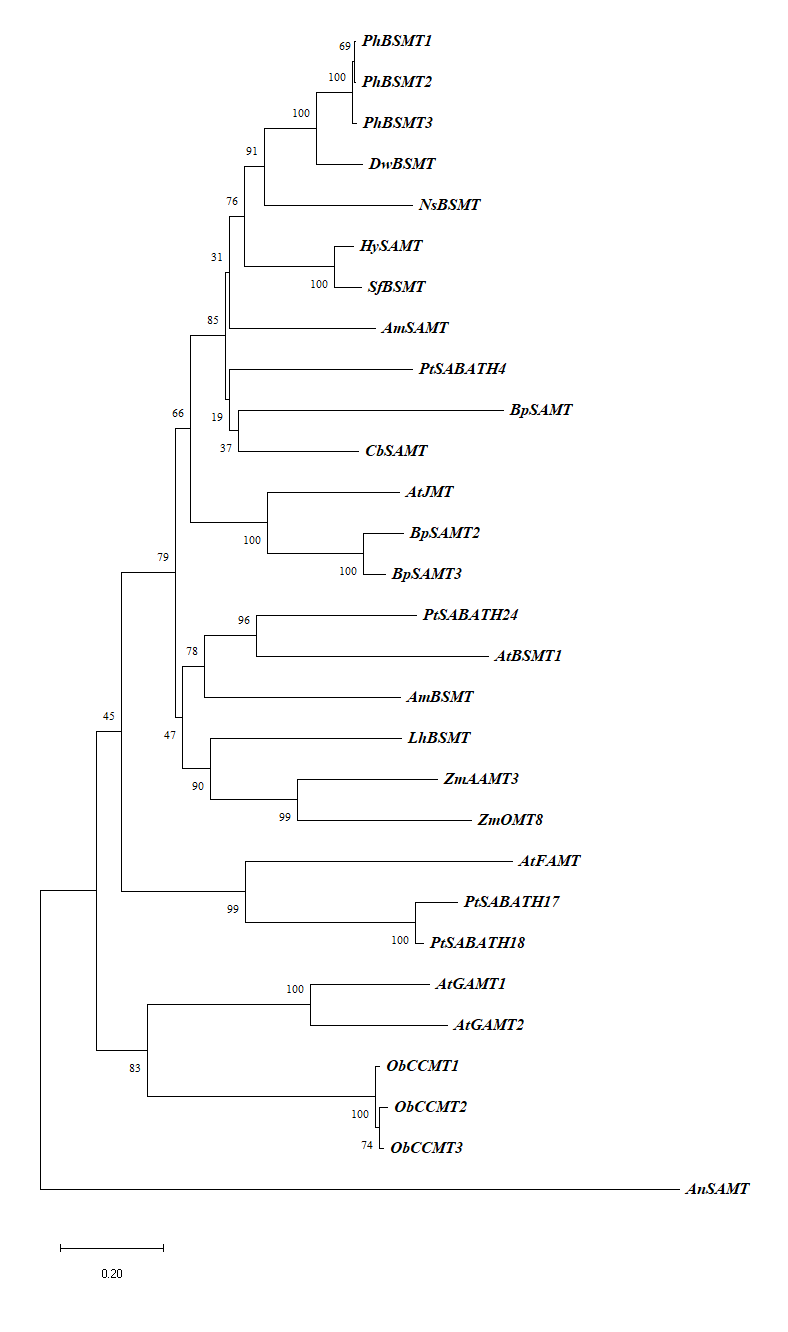


**S Fig 1**: The phylogenetic tree was constructed to identify a *SAMT* homologous gene as a candidate gene in *B. pendula* using the maximum likelihood method in the MEGA X software (Kumar et al., 2018). Three hits with similar E-values emerged after the tBlastn search ((Bpev01.c0161.g0056.m0001 (*BpSAMT2*; E-value: 4E-47), Bpev01.c0161.g0057.m0001 (*BpSAMT3;* E-value: 9E-46) and Bpev01.c0425.g0055 (*BpSAMT;* E-value: 1E-45)), and were included to identify a most probable *SAMT* homologous gene in *B. pendula*. Of the three, the *BpSAMT* gene was chosen as a candidate since it formed a clade with previously functionally characterized *Clarkia breweri* *SAMT* (*CbSAMT*)*,* *P. hybrid BSMT* (*PhBSMT*)*, D. wrightii BSMT* (*DwBSMT*)*, A. majus SAMT* (*AmSAMT*) and *P. trichocarpa SAMT* (*PtSABATH4*). The *BpSAMT2* and *BpSAMT3* were excluded from the analysis since they formed a distinct clade with functionally characterized *Arabidopsis* methyl jasmonate (*AtJMT*). Therefore, *BpSAMT* was selected as a candidate gene for further analysis. *SAMT* from the fungus *Aspergillus niger* (NT166520) was used as an outgroup species. The numbers at the nodes indicate bootstrap values calculated with 1,000 replicates. Branches are drawn to scale with the bar indicating 0.20 substitutions per site.

**
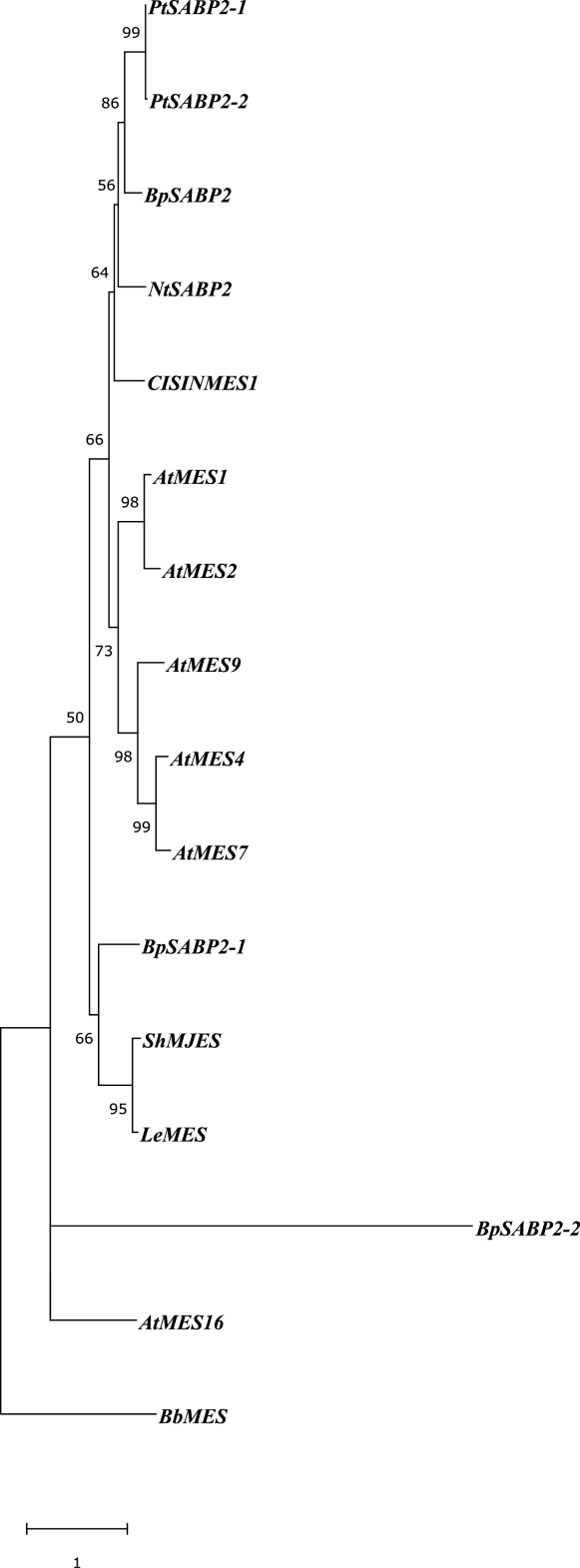
**

**S Fig 2**: The phylogenetic tree was constructed to identify a *SABP2* homologous gene as a candidate gene in *B. pendula* using the maximum likelihood method in the MEGA X software (Kumar et al., 2018). Three hits with similar E-values emerged after the tBlastn search (Bpev01.c0161.g0056.m0001 (*BpSABP2*; E-value: 7E-46) Bpev01.c0161.g0057.m0001 (*BpSABP2-2*; E-value: 6E-36) and Bpev01.c0425.g0055.m0001 (*BpSABP2-3*; E-value: 6E-35)), and were included to identify a most probable *SABP2* homologous gene in *B. pendula*. Of the three, the *BpSABP2* gene was chosen as a candidate since it formed a clade with previously functionally characterized *P. trichocarpa* SABP2 (*PtSABP2-1, PtSABP2-2*) and *N. tobacco* *SABP2* (*NtSABP2*). The *BpSABP2-2* and *BpSABP2-3* were excluded from the analysis since they formed a distinct clade. Therefore, *BpSABP2* was selected as a candidate gene for further analysis. The *MES* from *Beauveri abassiana* (PMB68924.1) was used as an outgroup species. The numbers at the nodes indicate bootstrap values calculated with 1,000 replicates. Branches are drawn to scale with the bar indicating 1 substitution per site.

**
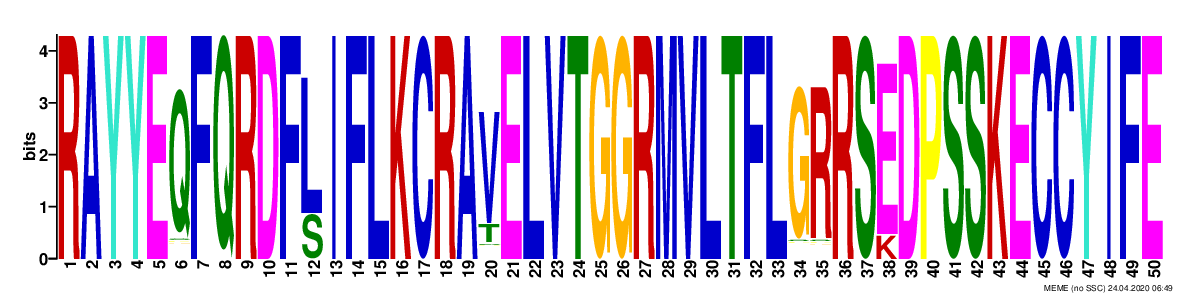
**

**
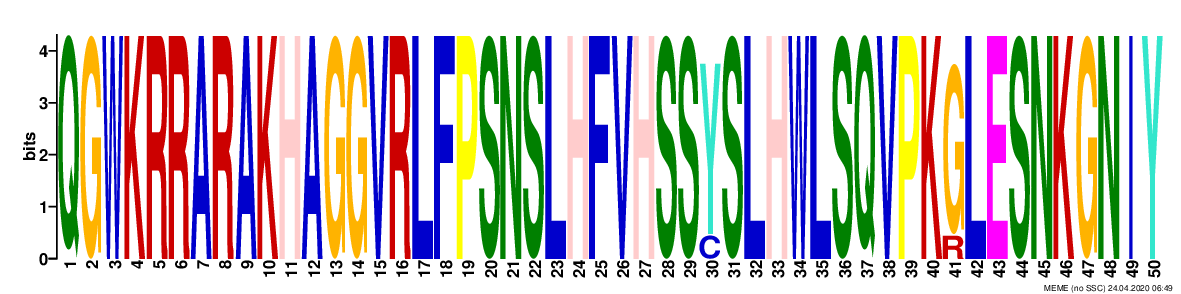
**

**
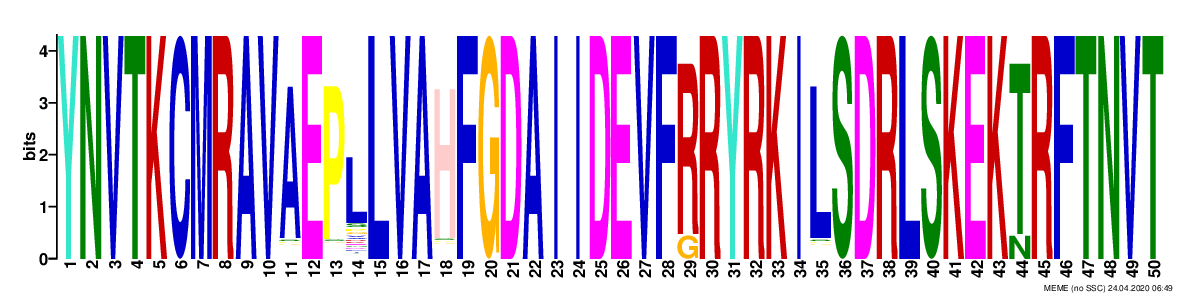
**

**
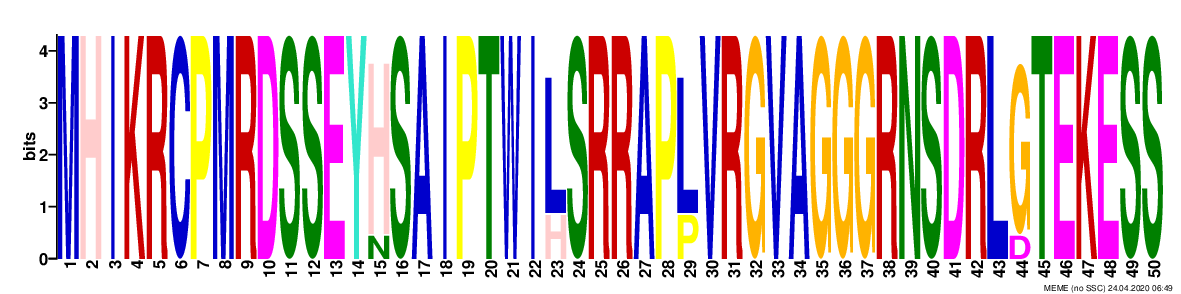
**

**
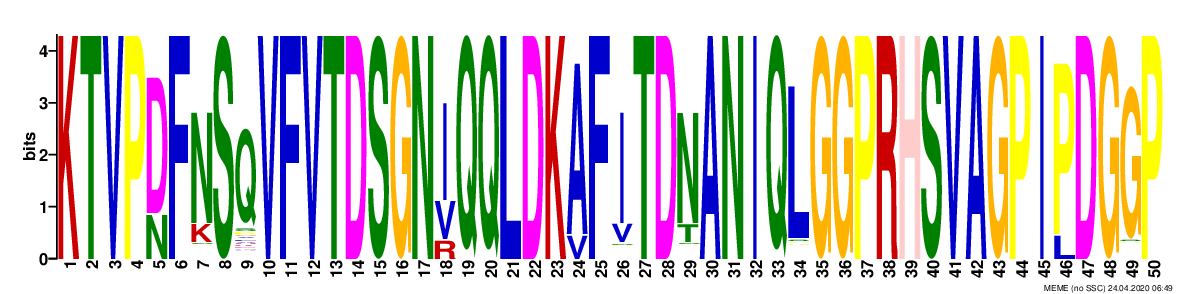
**

**
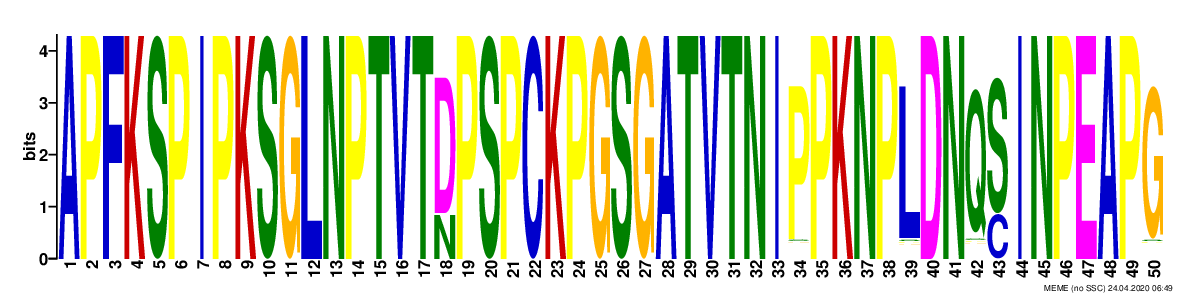
**

**
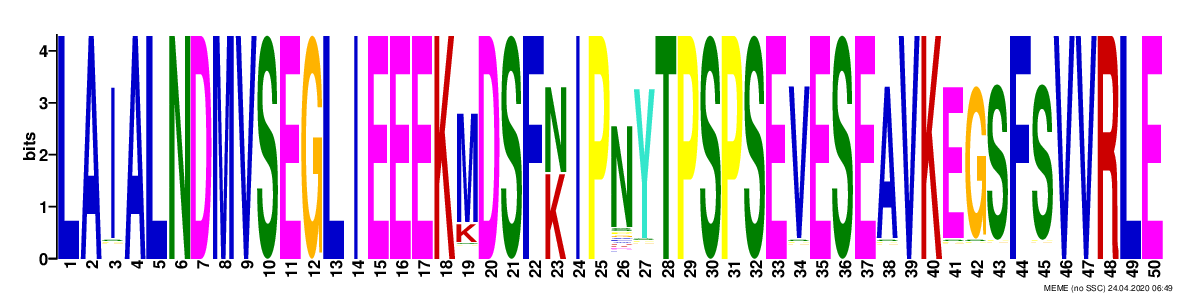
**

**
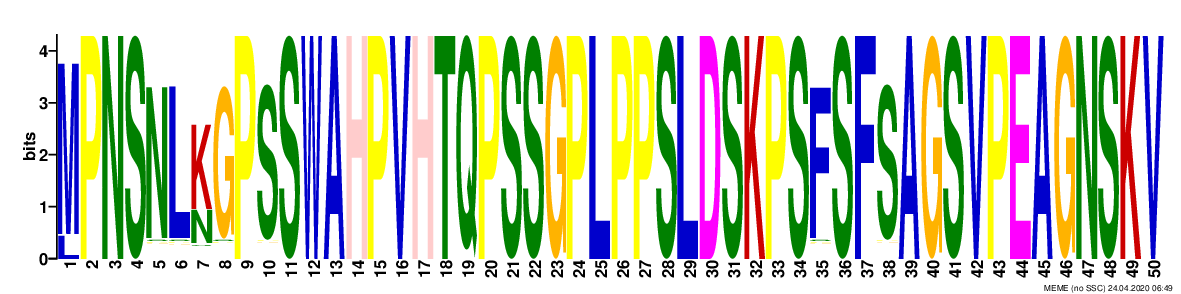
**

**
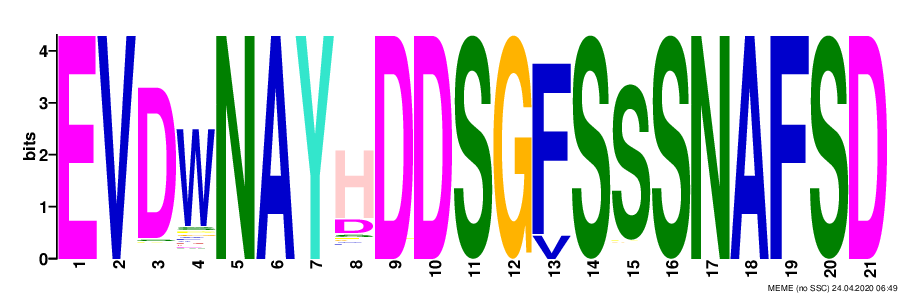
**

**
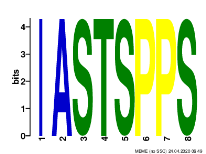
**

**
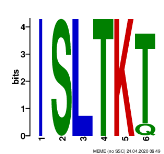
**

**S Fig 3 (A):** Motif logos observed in the *Betula* SAMT amino acid sequences.

**
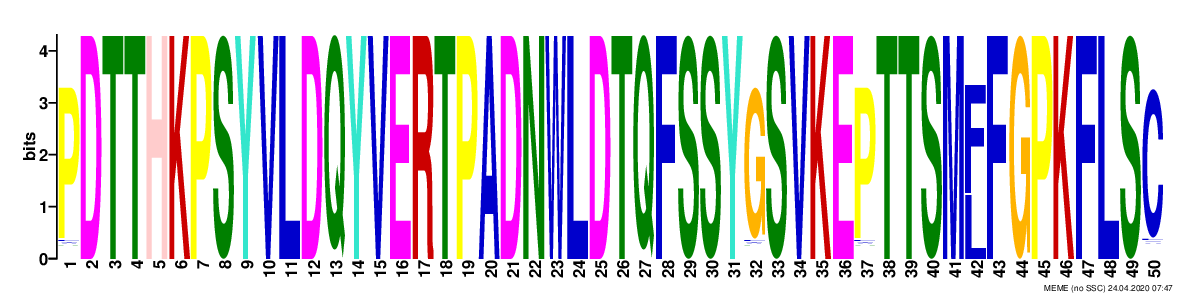
**

**
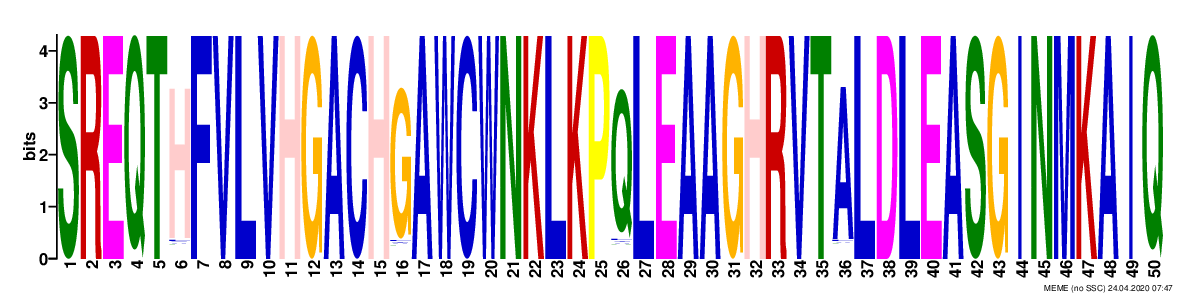
**

**
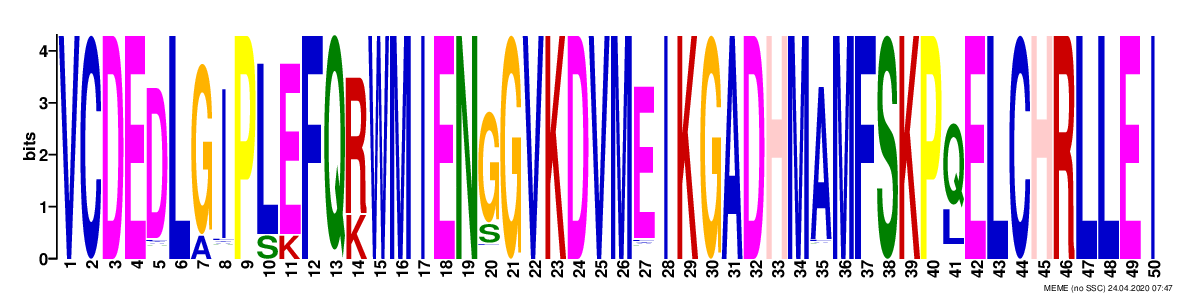
**

**
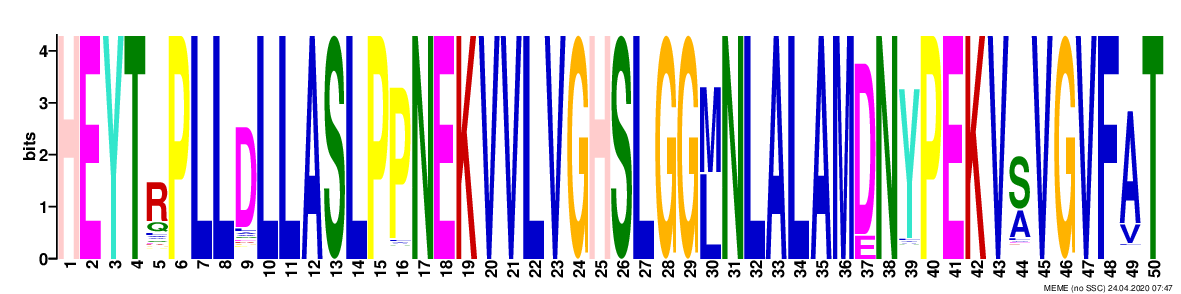
**

**
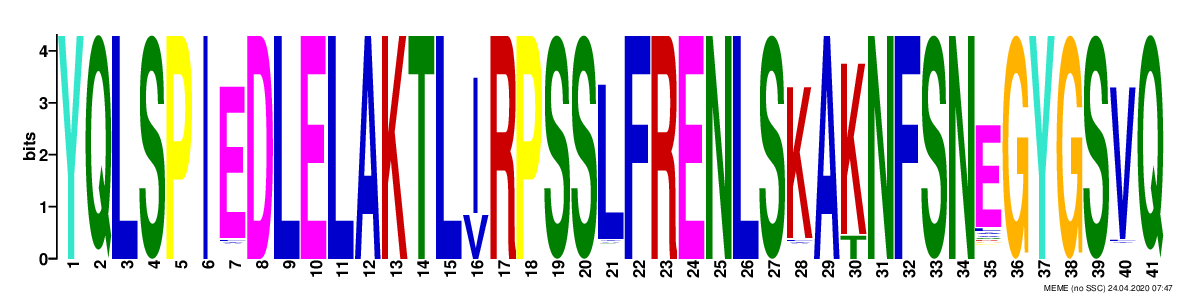
**

**S Fig 3 (B):** Motif logos observed in the *Betula* SABP2 amino acid sequences.


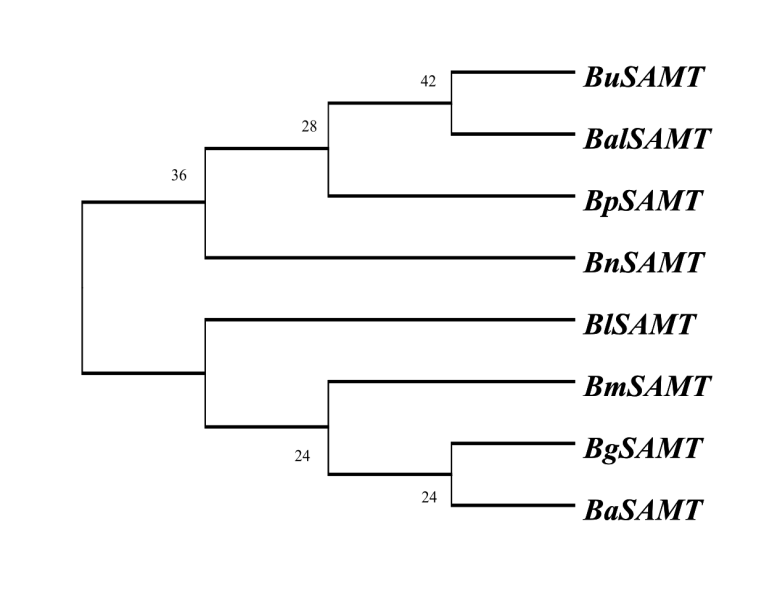

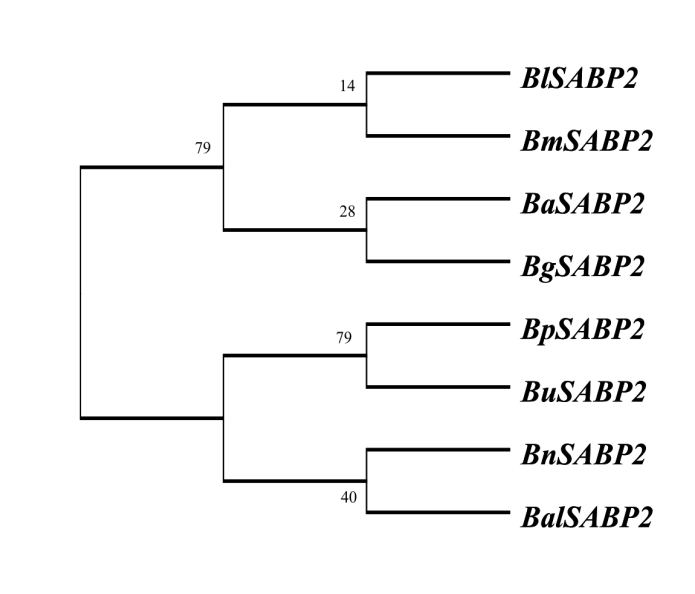


(A)

(B)

**S Fig 4:** Evolutionary relationship of (A) *SAMT* and (B) *SABP2* in eight high and low MeSA-producing *Betula* species: the phylogenetic tree was constructed using the maximum likelihood method in the MEGA X software (Kumar et al., 2018) using *SAMT* and *SABP2* amplified from *B. lenta* (Bl), *B. alleghaniensis* (Ba), *B. grossa* (Bg), *B. medwediewii* (Bm), *B. pendula* (Bp), *B. utilis* (Bu), *B. nana* (Bn) and *B. alnoides* (Bal) species. Exon regions of *SAMT* (A) and *SABP2* (B) were sequenced from all the *Betula* species and converted into the amino acid sequences. The numbers at the nodes indicate bootstrap values calculated for 1,000 replicates.

**S Table 1:** Search results obtained after the BLAST uing *C. breweri* SAMT (CbSAMT, AF133053) (Ross et al., 1999) and *N. tabacum* SABP2 (NtSABP2, AY485932) (Kumar et al., 2003) amino acid sequences as a reference.

| **Query** | **Chr** | **Position** | **HSP No.** | **Length** | **E-value** | **Percent ID** | **Score** | **Feature ID** | **Closest Feature** |
| --- | --- | --- | --- | --- | --- | --- | --- | --- | --- |
| AF133053 | Bpe_Chr9 | 3382995 | 1 | 239 | 4E-47 | 41 | 178 | 1.24E+09 | [Bpev01.c0161.g0056.m0001](https://genomevolution.org/coge/FeatView.pl?accn=Bpev01.c0161.g0056.m0001) (BpSAMT2) |
|  | Bpe_Chr9 | 3392706 | 2 | 241 | 9E-46 | 39.8 | 174 | 1.24E+09 | [Bpev01.c0161.g0057.m0001](https://genomevolution.org/coge/FeatView.pl?accn=Bpev01.c0161.g0057.m0001) (BpSAMT3) |
|  | Bpe_Chr9 | 684525 | 3 | 144 | 1E-45 | 61.8 | 174 | 1.24E+09 | [Bpev01.c0425.g0055.m0001](https://genomevolution.org/coge/FeatView.pl?accn=Bpev01.c0425.g0055.m0001) (BpSAMT) |
| AY485932 | Bpe_Chr5 | 26048646 | 1 | 139 | 7E-46 | 55.3 | 171 | 1.24E+09 | [Bpev01.c0015.g0219.m0001](https://genomevolution.org/coge/FeatView.pl?accn=Bpev01.c0015.g0219.m0001) (BpSABP2) |
|  | Bpe_Chr5 | 26029215 | 2 | 123 | 6E-36 | 48.7 | 142 | 1.24E+09 | [Bpev01.c0015.g0217.m0001](https://genomevolution.org/coge/FeatView.pl?accn=Bpev01.c0015.g0217.m0001) (BpSABP2-2) |
|  | Bpe_Chr5 | 26039541 | 3 | 130 | 6E-35 | 47.6 | 139 | 1.24E+09 | [Bpev01.c0015.g0218.m0001](https://genomevolution.org/coge/FeatView.pl?accn=Bpev01.c0015.g0218.m0001) (BpSABP2-3) |

**S Table 2:** SABP2 and SAMT amino acid sequences used from different species for phylogenetic analysis and their gene bank accession numbers.

| **Amino acid sequence SABP2** | **Species** | **Accession number** |
| --- | --- | --- |
| PtSABP2-1 | *P. trichocarpa* | estExt-fgenesh4_pm.C_LG_VII0354 |
| PtSABP2-2 | *P. trichocarpa* | eugene3.00070971 |
| NtSABP2 | *N. tabacum* | AY485932 |
| AtMES1 | *A. thaliana* | At2g23620 |
| AtMES2 | *A. thaliana* | At2g23600 |
| AtMES4 | *A. thaliana* | At2g23580 |
| AtMES7 | *A. thaliana* | At2g23560 |
| AtMES9 | *A. thaliana* | At4g37150 |
| AtMES16 | *A. thaliana* | At4g16690 |
| StMJE | *S. tuberosum* | NP001275411.1 |
| LeMES | *L. esculentum* | AY455313 |
| Citrus MES1CISIN | *C. sinensis* | KDO79352 |
| BbSABP2* | *Beauveria bassiana* | PMB68924.1 |
| PtSABATH4 | *P. trichocarpa* | Potri.007G021300 |
| PtSABATH17 | *P. trichocarpa* | Potri.017G122700 |
| PtSABATH18 | *P. trichocarpa* | Potri.017G122900 |
| PtSABATH24 | *P. trichocarpa* | Potri.019G022400 |
| AtSABATH2 | *A. thaliana* | AT1G19640 |
| AtSABATH8 (AtBSMT1) | *A. thaliana* | AT3G11480 |
| AtSABATH11 | *A. thaliana* | AT3G44860 |
| AtSABATH13 | *A. thaliana* | AT4G26420 |
| AtSABATH23 | *A. thaliana* | AT5G56300 |
| CbSAMT | *Clarkia breweri* | AF133053 |
| ObCCMT1 | *Ocimum basilicum* | EU033968 |
| ObCCMT2 | *Ocimum basilicum* | EU033969 |
| ObCCMT3 | *Ocimum basilicum* | EU033970 |
| ZmAAMT2 | *Zea mays* | HM242246 |
| ZmAAMT3 | *Zea mays* | HM242247 |
| ZmOMT8 | *Zea mays* | HM242248 |
| LhBSMT | *Lilium hybrid Yellowen* | KJ755672 |
| *PhBSMT1* | *Petunia hybrid* | AAO45012 |
| *PhBSMT2* | *Petunia hybrid* | AAO45013 |
| *PhBSMT3* | *Petunia hybrid* | ABF50941 |
| *DwBSMT* | *Datura wrightii* | ABO71015 |
| *NsBSMT* | *Nicotiana suaveolens* | ACZ55217.1 |
| *Nicotiana suaveolens BSMT* | *Nicotiana suaveolens* | CAF31508.1 |
| *HcSAMT* | *Hoya carnosa* | CAI05934 |
| *ShBSMT* | *Stephanotis floribunda* | CAC33768.1 |
| *AmSAMT* | *Antirrhinum majus* | AAN40745.1 |
| *AmBSMT* | *Antirrhinum majus* | AAF98284.1 |
| *AnSAMT** | *Aspergillus niger* | NT_166520 |

*Outgroup species

**S Table 3:** List of the PCR primers used for the candidate gene amplification and qRT-PCR analysis in the present study.

| **Regions** | **Numbers** | **Primer sequence (5’ to 3’)** | **T_a_** |
| --- | --- | --- | --- |
| *SABP2*-E1_F | 2687 | GGCAGCTTCAAGAGAGCAAA | 57 |
| *SABP2*-E1_R | 2573 | TGGGGTCCTCTCAACATACT | 53 |
| *SABP2*-E2_F | 2663 | AGTATGTTGAGAGGACCCCA | 55 |
| *SABP2*-E2_R | 2622 | ACTAAAGTCTTGGCCAGCTC | 54 |
| *SABP2*-E3_F | 2623 | GAGCTGGCCAAGACTTTAGT | 54 |
| *SABP2*-E3_R | 2664 | TTAAGCGTATTTGCGTGCAA | 57 |
| *SABP2_*UTR_F | 2869 | CAGGCTGTCATGAAATAGTAGT | 55 |
| *SABP2_*UTR_R | 2602 | GCCCCATGCACTAGAACAAAAT | 57 |
| *SAMT_*UTR_F | 2613 | TTCCATCTACTGCCGAATCC | 54 |
| *SAMT_UTR_R* | 2614 | CTCTTTGACATTGGCTGCGT | 56 |
| *SAMT_UTR+E1_F* | 2605 | AGTTCTGTGAAGCAAGTTCC | 55.3 |
| *SAMT_UTR+E1_R* | 2606 | GAAGAGTCTCTCATTGGGCA | 57.3 |
| *SAMT_E2_F* | 2569 | TCAGCCGTAGAGCACCACTG | 60 |
| *SAMT_E2_R* | 2744 | CCTGACGCCCCCTGCATGCT | 63 |
| *SAMT_E3_F* | 2772 | CGTCAGGCTTTTTCCGAGCA | 58 |
| *SAMT_E3_R* | 2787 | GTAGTAAGCCCGAAGCACGC | 58 |
| *SAMT_E4_F* | 2625 | GCGTGCTTCGGGCTTACTAC | 61 |
| *SAMT_E4_R* | 2630 | CCTCGGAGACCATGTCATTG | 58 |
| *SAMT_E5_F* | 2633 | CTCCGAGGGCCTCATAGAAG | 56 |
| *SAMT_E5_R* | 2688 | TCATCCTGTTTTAGTCAAGG | 53 |
| SABP2-qPCR_F | 2937 | CGGCCTTTGTTGGATCTTCTGG | 59.6 |
| SABP2-qPCR_R | 2871 | GAAAACCCCAACGGATACCT | 58.3 |
| SAMT-qPCR_F | 2949 | ACACGCCATCCCCATCAGAA | 59.4 |
| SAMT-qPCR_R | 2976 | GCATTGGACGAGCTGAATCC | 59.4 |
| *Ubiquitin*_F | 2960 | CCAGAGGATCCACAGTGCTA | 59.4 |
| *Ubiquitin*_R | 2961 | GAGAAGCCGATCTGCAATGG | 59.4 |
| Actin_F | 2958 | CGGATGAGCAAGGAGATCAC | 59.4 |
| Actin_R | 2959 | CACATCTGCTGGAAGGTGCT | 59.4 |
| SABP2_SNP-F | 3009 | GAACAAGCTCAAACCACAGC | 56 |
| SABP2_SNP-F | 3010 | GATCCAAGACATATGATGGC | 56 |

**S Table 4:** GenBank accession numbers of *SAMT* and *SABP2* genes analyzed in different *Betula* species as well as the sequences used in the phylogenetic analysis.

| *Betula* gene | Accession number | CDS sequence length | AA sequence length |
| --- | --- | --- | --- |
| *B. pendula (BpSAMT)* | MT647740 | 1344 | 447 |
| *B. alleghaniensis (BaSAMT)* | MT647741 | 1344 | 447 |
| *B. lenta (BlSAMT)* | MT647742 | 1344 | 447 |
| *B. grossa (BgSAMT)* | MT647743 | 1344 | 447 |
| *B. alnoides  (BalSAMT)* | MT647744 | 1344 | 447 |
| *B. utilis (BuSAMT)* | MT647745 | 1347 | 447 |
| *B. medwediewii (BmSAMT)* | MT647746 | 1347 | 447 |
| *B. nana (BnSAMT)* | MT647747 | 1348 | 447 |
| *B. pendula (BpSABP2)* | MT606169 | 792 | 263 |
| *B. alleghaniensis (BaSABP2)* | MT606170 | 792 | 263 |
| *B. lenta (BlSABP2)* | MT606171 | 792 | 263 |
| *B. grossa (BgSABP2)* | MT606172 | 792 | 263 |
| *B. alnoides  (BalSABP2)* | MT606173 | 792 | 263 |
| *B. utilis (BuSABP2)* | MT606174 | 792 | 263 |
| *B. medwediewii (BmSABP2)* | MT606175 | 1347 | 263 |
| *B. nana (BnSABp2)* | MT606176 | 1348 | 263 |
| *Betula* Ubiquitin exon-8 | MT602551 | 2778 | - |

**S Table 5:** Comparative analysis of SAMT and SABP2 amino acid sequences.

| **Query** | **Subject** | **Coverage** | **Identity** |
| --- | --- | --- | --- |
| BpSAMT | CbSAMT | 55% | 63.7% |
| BpSAMT | AtBSMT1 | 50% | 39.9% |
| BaSAMT | BpSAMT | 100% | 95.5% |
| BpSAMT | PtSABATH4 | 51% | 54.37% |
| BalSAMT | BpSAMT | 100% | 99.6% |
| BlSAMT | BpSAMT | 100% | 96.9% |
| BmSAMT | BpSAMT | 100% | 97.5% |
| BuSAMT | BpSAMT | 100% | 93.5% |
| BnSAMT | BpSAMT | 100% | 95.9% |
| BgSAMT | BpSAMT | 99% | 97.53% |
| BpSABP2 | NtSABP2 | 98% | 64.73% |
| BpSABP2 | PtSABP2-1 | 9% | 28.6% |
| BpSABP2 | PtSABP2-2 | 4% | 40.9% |
| BpSABP2 | AtMES9 | 96% | 53.5% |
| BaSABP2 | BpSABP2 | 100% | 93.2% |
| BalSABP2 | BpSABP2 | 100% | 96.2% |
| BlSABP2 | BpSABP2 | 100% | 95.1% |
| BmSABP2 | BpSABP2 | 100% | 98.1% |
| BuSABP2 | BpSABP2 | 100% | 94.7% |
| BnSABP2 | BpSABP2 | 100% | 97.2% |
| BgSABP2 | BpSABP2 | 100% | 93.5% |

**S Table 6:** List of *Betula* species from different botanical gardens (BG) that were used to validate the detected nucleotide substitutions.

| **Species Name** | **Place of sample origin** | **Distribution** | **2n** | **Subgenus** | **Section** | **Accession number** |
| --- | --- | --- | --- | --- | --- | --- |
| *B. alleghaniensis* | BG Tharandt, Germany | North America | 6n | *Aspera* | *Lentae* | MT591530 |
| *B. alleghaniensis* | NW-FVA*, Germany | North America | 6n | *Aspera* | *Lentae* | MT591531 |
| *B. alleghaniensis* | BG Giessen, Germany | North America | 6n | *Aspera* | *Lentae* | MT591532 |
| *B. alleghaniensis* | Bochum | North America | 6n | *Aspera* | *Lentae* | MT591533 |
| *B. alleghaniensis* | Bonn | North America | 6n | *Aspera* | *Lentae* | MT591534 |
| *B. alleghaniensis* | Frankfurt | North America | 6n | *Aspera* | *Lentae* | MT591535 |
| *B. lenta* | BG Giessen, Germany | North America | 2n | *Aspera* | *Lentae* | MT591536 |
| *B. lenta* | BG Tharandt, Germany | North America | 2n | *Aspera* | *Lentae* | MT591537 |
| *B. lenta* | BG Marburg, Germany | North America | 2n | *Aspera* | *Lentae* | MT591538 |
| *B. lenta* | Bochum | North America | 2n | *Aspera* | *Lentae* | MT591539 |
| *B. lenta* | Frankfurt | North America | 2n | *Aspera* | *Lentae* | MT591540 |
| *B. lenta* | Dresden | North America | 2n | *Aspera* | *Lentae* | MT591541 |
| *B. grossa* | BG Tharandt, Germany | Japan | 12n | *Aspera* | *Lentae* | MT591542 |
| *B. grossa* | Bonn | Japan | 12n | *Aspera* | *Lentae* | MT591543 |
| *B. grossa* | Bayreuth | Japan | 12n | *Aspera* | *Lentae* | MT591544 |
| *B. grossa* | Osnabrück | Japan | 12n | *Aspera* | *Lentae* | MT591545 |
| *B. medwediewii* | BG Liverpool, Great Britain | Caucasus Mountains | 10n | *Aspera* | *Lentae* | MT572373 |
| *B. medwediewii* | Bonn | Caucasus Mountains | 10n | *Aspera* | *Lentae* | MT572374 |
| *B. medwediewii* | Hamburg | Caucasus Mountains | 10n | *Aspera* | *Lentae* | MT572375 |
| *B. medwediewii* | Marburg | Caucasus Mountains | 10n | *Aspera* | *Lentae* | MT572376 |
| *B. pendula* | BG Grosshansdorf, Germany | Europe and East Asia | 2n | *Betula* | *Betula* | MT591546 |
| *B. pendula* | BG Grosshansdorf, Germany | Europe and East Asia | 2n | *Betula* | *Betula* | MT591547 |
| *B. pendula* | BG Grosshansdorf, Germany | Europe and East Asia | 2n | *Betula* | *Betula* | MT591548 |
| *B. pendula* | BG Grosshansdorf, Germany | Europe and East Asia | 2n | *Betula* | *Betula* | MT591549 |
| *B. pendula* | BG Grosshansdorf, Germany | Europe and East Asia | 2n | *Betula* | *Betula* | MT591550 |
| *B. pendula* | ReinkeBaumschulen | Europe and East Asia | 2n | *Betula* | *Betula* | MT591551 |
| *B. utilis* | Kiel (private), Germany | Himalayas | 4n | *Betula* | *Costatae* | MT591552 |
| *B. utilis* | Gottingen | Himalayas | 4n | *Betula* | *Costatae* | MT591553 |
| *B. utilis* | weltwald-harz | Himalayas | 4n | *Betula* | *Costatae* | MT591554 |
| *B. utilis* | Hamburg | Himalayas | 4n | *Betula* | *Costatae* | MT591555 |
| *B. nana* | BG Montreal, Canada | Arctic region | 2n | *Betula* | *Apterocaryon* | MT591556 |
| *B. nana* | Munchen | Arctic region | 2n | *Betula* | *Apterocaryon* | MT591557 |
| *B. nana* | Belgium | Arctic region | 2n | *Betula* | *Apterocaryon* | MT591558 |
| *B. nana* | Cambridge | Arctic region | 2n | *Betula* | *Apterocaryon* | MT591559 |
| *B. alnoides* | BG Tharandt, Germany | India, Bhutan, Nepal, China | 2n | *Acuminata* | *Acuminatae* | MT591560 |
| *B. alnoides* | BG Tharandt, Germany | India, Bhutan, Nepal, China | 2n | *Acuminata* | *Acuminatae* | MT591561 |
| *B. alnoides* | Eberswalde | India, Bhutan, Nepal, China | 2n | *Acuminata* | *Acuminatae* | MT591562 |
| *B. alnoides* | BGTharandt | India, Bhutan, Nepal, China | 2n | *Acuminata* | *Acuminatae* | MT591563 |

**S Table 7:** Pfam domain analysis of *Betula* *SAMT* and *SABP2* compared to homologous genes in *C. breweri*, *P. trichocarpa* and *N. tabacum.*

| **Query** | **Pfam** | **Position (Independent E-value)** | **Description** |
| --- | --- | --- | --- |
| *CbSAMT* | [Methyltransf_7](https://www.genome.jp/dbget-bin/www_bget?pf:Methyltransf_7) | 39..356(2.9e-117) | PF03492, SAM-dependent carboxyl methyltransferase |
| *PtSABATH4* | [Methyltransf_7](https://www.genome.jp/dbget-bin/www_bget?pf:Methyltransf_7) | 38..252(2.5e-86) 276..320(5e-13) 318..362(2.7e-06) | PF03492, SAM-dependent carboxyl methyltransferase |
| *BlSAMT* | [Methyltransf_7](https://www.genome.jp/dbget-bin/www_bget?pf:Methyltransf_7) | 220..445(6.1e-85) | PF03492, SAM-dependent carboxyl methyltransferase |
| *BaSAMT* | [Methyltransf_7](https://www.genome.jp/dbget-bin/www_bget?pf:Methyltransf_7) | 220..445(1e-76) | PF03492, SAM-dependent carboxyl methyltransferase |
| *BgSAMT* | [Methyltransf_7](https://www.genome.jp/dbget-bin/www_bget?pf:Methyltransf_7) | 220..445(5.7e-81) | PF03492, SAM-dependent carboxyl methyltransferase |
| *BmSAMT* | [Methyltransf_7](https://www.genome.jp/dbget-bin/www_bget?pf:Methyltransf_7) | 220..445(2.7e-80) | PF03492, SAM-dependent carboxyl methyltransferase |
| *BpSAMT* | [Methyltransf_7](https://www.genome.jp/dbget-bin/www_bget?pf:Methyltransf_7) | 220..445(5.5e-83) | PF03492, SAM-dependent carboxyl methyltransferase |
| *BuSAMT* | [Methyltransf_7](https://www.genome.jp/dbget-bin/www_bget?pf:Methyltransf_7) | 220..445(1e-82) | PF03492, SAM-dependent carboxyl methyltransferase |
| *BnSAMT* | [Methyltransf_7](https://www.genome.jp/dbget-bin/www_bget?pf:Methyltransf_7) | 220..445(2.7e-83) | PF03492, SAM-dependent carboxyl methyltransferase |
| *BalSAMT* | [Methyltransf_7](https://www.genome.jp/dbget-bin/www_bget?pf:Methyltransf_7) | 220..445(4.1e-84) | PF03492, SAM-dependent carboxyl methyltransferase |
| *NtSABP2* | [Abhydrolase_6](https://www.genome.jp/dbget-bin/www_bget?pf:Abhydrolase_6) | 7..245(4.6e-20) | PF12697, Alpha/beta hydrolase family |
| *PtSABP2-1* | [Abhydrolase_6](https://www.genome.jp/dbget-bin/www_bget?pf:Abhydrolase_6) | 10..251(7.8e-22) | PF12697, Alpha/beta hydrolase family |
| *PtSABP2-2* | [Abhydrolase_6](https://www.genome.jp/dbget-bin/www_bget?pf:Abhydrolase_6) | 10..251(1.4e-19) | PF12697, Alpha/beta hydrolase family |
| *BlSABP2* | [Abhydrolase_6](https://www.genome.jp/dbget-bin/www_bget?pf:Abhydrolase_6) | 10..251(1.7e-19) | PF12697, Alpha/beta hydrolase family |
| *BaSABP2* | [Abhydrolase_6](https://www.genome.jp/dbget-bin/www_bget?pf:Abhydrolase_6) | 10..251(9.9e-16) | PF12697, Alpha/beta hydrolase family |
| *BgSABP2* | [Abhydrolase_6](https://www.genome.jp/dbget-bin/www_bget?pf:Abhydrolase_6) | 10..251(7.2e-17) | PF12697, Alpha/beta hydrolase family |
| *BmSABP2* | [Abhydrolase_6](https://www.genome.jp/dbget-bin/www_bget?pf:Abhydrolase_6) | 10..251(2.7e-17) | PF12697, Alpha/beta hydrolase family |
| *BpSABP2* | [Abhydrolase_6](https://www.genome.jp/dbget-bin/www_bget?pf:Abhydrolase_6) | 10..251(6.5e-19) | PF12697, Alpha/beta hydrolase family |
| *BuSABP2* | [Abhydrolase_6](https://www.genome.jp/dbget-bin/www_bget?pf:Abhydrolase_6) | 10..251(1.2e-17) | PF12697, Alpha/beta hydrolase family |
| *BnSABP2* | [Abhydrolase_6](https://www.genome.jp/dbget-bin/www_bget?pf:Abhydrolase_6) | 10..251(7.2e-18) | PF12697, Alpha/beta hydrolase family |
| *BalSABP2* | [Abhydrolase_6](https://www.genome.jp/dbget-bin/www_bget?pf:Abhydrolase_6) | 10..251(9.7e-18) | PF12697, Alpha/beta hydrolase family |

**S Table 8:** The DNA sequences of the *Betula* SAMT and SABP2 promoter regions as amplified by the primer walking method. The putative TATA box, CAAT box and other important cis-regulatory elements are marked out.

| **Promoter** | **Accession number** |
| --- | --- |
| *BpSABP2* | MT542185 |
| *BuSABP2* | MT542186 |
| *BaSABP2* | MT542187 |
| *BlSABP2* | MT542188 |
| *BmSABP2* | MT542189 |
| *BnSABP2* | MT542190 |
| *BalSABP2* | MT542191 |
| *BpSAMT* | MT542177 |
| *BuSAMT* | MT542178 |
| *BaSAMT* | MT542179 |
| *BgSAMT* | MT542180 |
| *BlSAMT* | MT542181 |
| *BmSAMT* | MT542182 |
| *BnSAMT* | MT542183 |
| *BalSAMT* | MT542184 |

**S Table 9:** Comparative analysis of putative *cis*-acting regulatory elements identified in the promoter region of seven *Betula* SAMT using PlantCARE databases.

| **Promoter** | **Function** | ***BaSAMT***  **(604)** | ***BlSAMT***  **(628)** | ***BuSAMT***  **(607)** | ***BalSAMT***  **(606)** | ***BpSAMT***  **(1000)** | ***BnSAMT***  **(598)** | **BmSAMT**  **(596)** | ***BgSAMT***  **(598)** |
| --- | --- | --- | --- | --- | --- | --- | --- | --- | --- |
| TATA box | core promoter element around -30 of transcription start | 311-TATA | 338-TATA  477-TATA | 314-TATA | 313-TATA | 697-TATA | 300- TATA | 301-TATA | 320- TATA |
| CAAT box | common cis-acting element in promoter and enhancer regions | 62-CAAAT  277+CCAAT  106-CAAT  278+CAAT  84-CCAAT  230-CAAT  158+CAAT  571+CAAT | 12-CAAT  304+CCAAT  111- CCAAT  595+CAAT  80-CAAT  346-CAAAT  185+CAAT  305+CAAT  53-CAAAT  530-CCAAT  133-CAAT  89-CAAAT  257-CAAT | 29-CAAAT  233-CAAT  161+CAAT  281+CAAT  87-CCAAT  280+CCAAT  146-CAAAT  574+CAAT  65-CAAAT  109-CAAT | 28-CAAAT  279+CAAAT  232-CAAT  573+CAAT  86-CCAAT  280+CAAT  160+CAAT  64-CAAAT  108-CAAT | 10+ CCAAT  412- CAAAT  211+ CCAAT  663+ CAAT  128- CAAAT  616- CAAT  299- CAAT  959+ CAAT  70- CAAT  470- CAAAT  268- CAAAT  664+ CAAT  181+ CCAAT  544+ CAAT  352+ CAAAT  448- CAAT  40+ CAAT  492- CCAAT  97- CCAAT | 15-CAAAT  266+CAAAT  147+CCAAT  561+CCAAT  73-CAAT  267+CAAT  219-CAAAT  51-CCAAT  95-CAAT | 16-CAAAT  267+CCAAT  220-CAAT  274+CAAAT  74+CAAAT  268+CCAAT  148+CAAT  561+CAAAT  52-CCAAT  96-CCAAT | 17- CAAAT  221- CAAT  149+ CAAT  310-CAAAT  75- CCAAT  269+ CAAT  179+ CAAAT  556+ CAAT  53- CAAAT  97- CAAT |
| ARE | cis-acting regulatory element essential for the anaerobic induction | 345-AAACCA | 372-AAACCA  522+AAACCA | 348-AAACCA | 347-AAACCA | 340-AAACCA | - | 335- AAACCA | - |
| A-Box | cis-acting regulatory element | - | 243+CCGTCC | - | - | - | - | - | - |
| AuxRR-core | cis-acting regulatory element involved in auxin responsiveness | - | 355-GGTCCAT | - | - | 714-GGTCCAT | 217-GGTCCAT | 318- GGTCCAT | - |
| Circadian | cis-acting regulatory element involved in circadian control | - | 139-CAAAGATATC | - | - | - | - | - | - |
| GTGGC-motif | part of a light responsive element | - | - | - | - | 170-GATTCTGTGGC | - | - | - |
| chs-CMA2a | part of a light responsive element | - | - | - | - | 75-TCACTTGA  246- TCACTTGA | - | - | - |
| [AAGAA-motif](http://bioinformatics.psb.ugent.be/webtools/plantcare/cgi-bin/show_site_info.htpl?QWhere=ID_of_Site%20like%20%27GAAAGAA%27&StartAt=0&NbRecs=10) | - | 251- GAAAGAA | 278-GAAAGAA | 254- GAAAGAA | 253-GAAAGAA | 637- GAAAGAA | 240- GAAAGAA | 241- GAAAGAA | 242- GAAAGAA |
| MYB | - | 237+ TAACCA | 246+TAACCA | 240+TAACCA | 239+TAACCA | 632+ TAACCA | 226+ TAACCA | 227+ TAACCA | 228+ TAACCA |
| MYC | - | - | 345+CATTTG | - | - | 10-CATTTG  211-CATTTG  352-CATTTG  165+CATTTG  40-CATTTG  181-CATTTG |  |  | 179- CATTTG  341- CATTTG  309+ CATTTG |
| STRE | - | 222-  AGGGG | 249-AGGGG | 225- AGGGG | 224-AGGGG | 108- AGGGG  538+ AGGGG | 141+ AGGGG  211- AGGGG | 212- AGGGG | 213. AGGGG |
| TCA | -2 | - | 91-TCATCTTCAT | 3+ TCATCTTCAT | - | 108- TCATCTTCAT | - | - | - |
| Unnamed_4 | - | 132- CTCC  330- CTCC  184+ CTCC | 3 +  CTCC  159-  CTCC | 135-CTCC  333-CTCC  178+CTCC | 132-CTCC  332- CTCC  186+ CTCC | 362+  CTCC  581 -  CTCC | 121- CTCC  359+ CTCC | 122- CTCC  174+ CTCC | 123- CTCC  321- CTCC  175+ CTCC |
| ERE | - | - | - | - | - | - | - | - | 477+ATTTCATA |
| CCGTCC motif | - | - | 243+ CCGTCC | - | - | - | - | - | - |

**S Table 10:** Comparative analysis of putative *cis*-acting regulatory elements identified in the promoter region of seven *Betula* SABP2 using PlantCARE databases.

| **Promoter** | **Function** | **BaSABP2**  **(761 bp)** | **BlSABP2**  **(770)** | **BuSABP2**  **(698)** | **BalSABP2**  **(778)** | **BpSABP2**  **(1000)** | ***BnSABP2***  **(777)** | ***BmSABP2***  **(764)** |
| --- | --- | --- | --- | --- | --- | --- | --- | --- |
| TATA box | core promoter element around -30 of transcription start | 34+TATA  716-TATATA  606-CCTATAAAAA  718-TATA  714+ATTATA  717+ATATAA  715-TATATAA | 715+ ATTATA  718+ ATTATA  719- TATA  716- TATATAA  717- ATTATA | 83+ TATTTAAA  505- TATA  396- TATA  654+ ATATAA  345- ccTATAAAaa  652- TATATAA  414- TATA  655- TATA  262- ccTATAAAaa  651+ ATTATA  413+ ATTATA  653- ATTATA  395+ ATTATA  426- ATTATA | 23-TATAAAA  253-TATAA  68+TATATA  731+ATTATA  64-TATATAAATC  475-TATA  103+TATA  735-TATA  25-TATAA  254+TATA  101+ATTATA  733-TATATA  66-TATAAA  584-TATACA  102-TATAA  732-TATATAA  24-TATAAA  252+ATTATA  69+ATATAA  734+ATATAA  65-TATAAAT  505-TATTTAAA  70+TATA  474+ATATAA  26+TATA  586-TATA  67-TATATAA | 2+ TATA  1003- TATA  472- TATA  1002+ TATA  1- TATA  473- TATA  474- TATA  475+ TATA  513- TATA  514- TATA  515- TATA  516- TATA  517+ TATA  518+ TATA  519+ TATA  550+ TATA  551- TATA  552- TATA  744+ TATA  745- TATA  762+ TATA  763- TATA  775- TATA  852- TATA  854- TATA  999+ TATA  1000- TATA  1001- TATA | 147+ATTATA  482-TATA  351+TATA  731-TATATAA  350+ATTATA  730+ATTATA  471+TATTTAAA  733+ATATAA  148-TATAA  592-TATACA  410-TATTTAAA  732-TATATA  149+TATA  594-TATA  481+ATATAA  734-TATA | 352+ATATAA  514-TATTTAAA  441+TATA  719-ATATAA  417+TATTTAAA  582-TATA  483+ATATAA  721+ATATAA  402-TATTTAAA  580-TATA  473+TATTTAAA  720-TATA  418-ATATAA  718+TATA  448-TATTTAAA  722-TATA  353+TATA  401-ATATAA |
| CAAT box | common cis-acting element in promoter and enhancer regions | 110+CAAT  410-CCAAT  345+CAAAT  616+CAAT  123-CAAAT  497+CAAAT  386-CAAT  418-TGCCAAC  120+CAAAT  611-CCAAT  293-CAAT | 23+ CAAT  386- CAAT  120+ CAAAT  619- CAAT  60+ CAAAT  612-CAAT  293- CAAT  750+ CAAT  33+ CAAAT  418-CAAT  123- CAAAT  643+ CAAT  96- CAAAT  617+ CAAT  345+ CAAAT  757+ CAAT  9- CAAAT  410-CAAT  110+ CAAT  479+ CAAAT  36- CAAAT  147+ CAAAT | 94- CCAAT  466- CAAT  139+ CAAT  548- CAAT  138+ CAAT  541- CAAT  384+ CAAT  572+ CAAT  92- CCCAATTT  470+ CAAAT  301+ CAAT  546+ CAAT  137+ CCCAATTT  456- CAAAT | 98+CAAAT  535-CAAAT  249+CAAAT  627+CAAT  225-CAAAT  549+CAAAT  408+CAAT  653+CAAT  116+CAAT  545-CAAT  270+CAAAT  629-CAAT  159-CAAT  622-CCAAT  449+CAAT  74-CAAAT  131+CAAT | 19+ CAAT  283-CAAAT  147- CAAT  388+ CAAAT  47+ CCAAT  380- CAAT  240- CAAT  402+ CAAAT  21- CAAT  310+ CAAT  79- CAAT  433+ CAAAT  370+ CAAT  448+ CAAT  523- CAAT  547+ CAAT  568+ CAAAT  641+ CAAT  642+ CAAT  692+ CAAT  733+ CAAT  805- CAAT  815- CAAT  819+ CAAAT  890- CAAT  895+ CAAAT  897- CAAT  921+ CAAAT | 26+ CAAT  545- CAAT  144+ CCAAT  637- CAAAT  101+ CCAAT  630- CAAAT  317+ CAAT  661+ CAAAT  54- CAAT  559+ CCAAT  165+ CAAAT  635+ CAAAT  120- CCAAT  373+ CCAAT | 55-CAAT  491-CAAAT  320+CAAT  625-CAAT  135+CAAT  623+CAAT  375+CAAT  680+CAAT  121-CAAAT  145+CAAAT |
| ABRE | cis-acting element involved in the abscisic acid responsiveness | 16+CACGTG  17+ACGTG | - | 501- ACGTG | 671+ACGTG  670-CACGTG | 74+ ACGTG  191- CACGTG  938- CACGTG ACGTG  939+ CACGTG | - | - |
| Box 4 | part of a conserved DNA module involved in light responsiveness | 161+ATTAAT  326+ATTAAT  317+ATTAAT  330+ATTAAT | 326+ATTAAT  330+ ATTAAT  74+ ATTAAT  161+ ATTAAT  317+ ATTAAT | 452- ATTAAT | 283+ATTAAT  552-ATTAAT  531-ATTAAT | 143+ ATTAAT  801- ATTAAT  581- ATTAAT | 178+ ATTAAT  562- ATTAAT  541- ATTAAT | 539- ATTAAT |
| G-box | cis-acting regulatory element involved in light responsiveness | 16+CACGTG  508+CACGAC | - | 501- TACGTG | 670-CACGTG | 73- CACGTT  938- CACGTG  191+ CACGTG | - | - |
| GATA-motif | part of a light responsive element | 199-AAGGATAAGG | 199- AAGGATAAGG | 417+ AAGATAAGATT | 5-AAGATAAGATT  137- AAGATAAGATT | 454-, 766+  AAGATAAGATT | - | - |
| Sp1 | light responsive element | 651-GGGCGG | - | 514- GGGCGG | 596-GGGCGG | 863-GGGCGG | 603- GGGCGG | 591- GGGCGG |
| Circadian | cis-acting regulatory element involved in circadian control | - | - | - | 705+CAAAGATATC  696+ CAAAGATATC | 964+ CAAAGATATC  973+ CAAAGATATC | 704+ CAAAGATATC | - |
| GC-motif | enhancer-like element involved in anoxic specific indelibility | 230+CCCCCG | 230+ CCCCCG | - | - | - | - | - |
| P-box | gibberellin-responsive element | 266-CCTTTTG | 266- CCTTTTG | - | - | - | - | - |
| TCT-motif | part of a light responsive element | 363+TCTTAC | 363+ TCTTAC | - | - | - | - | - |
| TGA-element | auxin-responsive element | - | - | - | - | 62+AACGAC | - | - |
| Unnamed_1 | 60K protein binding site | - | 318- GAATTTAATTAA | 500- CGTGG | 31+ GAATTTAATTAA  672+ CGTGG  166+ GAATTTAATTAA | 480+GAATTTAATTAA | 61+ GAATTTAATTAA | - |
| Unnamed_2 | - | 231+CCCCGG | 231- CCCCGG | - | - | - | - | - |
| Unnamed_4 | - | 224+ CTCC  260- CTCC | 224+ CTCC | - | 348+ CTCC | 639+ CTCC | 247+ CTCC | 24+ CTCC |
| AT-TATA-Box | - | 716- TATATA | 717- TATATA | 653- TATATA | 733- TATATA  68+ TATATA  66- TATATAAA | 515- TATATAAA  1001- TATATA  517+ TATATA | 732- TATATA | 720- TATATA |
| STRE | - | 270+AGGGG  666+ AGGGG  655- AGGGG | 270+ AGGGG | 10- AGGGG  517- AGGGG  105+ AGGGG  77- AGGGG  117- AGGGG | - | 176- AGGGG  866- AGGGG | 606- AGGGG | 594- AGGGG |
| MYC | - | - | 664- CATGTG  617- CATGTG | 546- CAATTG | 687- CAATTG | 895- CAATTG | - | 623- CAATTG |
| W-box | - | - | 476- TTGACC | - | - | 80+ TTGACC | - | - |
| AAGAA-motif | - | - | - | - | 336- GAAAGAA | 509- GAAAGAA | 235- GAAAGAA  187- GAAAGAA | 228- GAAAGAA |
| ERE | - | - | - | 141- ATTTTAAA | 273+ ATTTTAAA | 27+ ATTTCATA  571+ ATTTCATA | 168+ ATTTTAAA  344- ATTTTAAA | 169+ ATTTTAAA  200- ATTTTAAA |
| WUN-Motif | wound-responsive element | - | - | 57+ AAATTTCCT  185+ AAATTTCCT | - | 289+ AAATTTCTT  823+ AAATTTCTT | 82+ AAATTTCTT | - |
| ATC-motif | part of a conserved DNA module involved in light responsiveness | - | - | - | - | - | 9+ AGTAATCT | - |
| MSA-like | cis-acting element involved in cell cycle regulation | - | - | - | - | - | 418 T/C)C(T/C)  AACGG  (T/C)(T/C)A | - |
| CGTCA-motif | cis-acting regulatory element involved in the MeJA-responsiveness | - | - | - | - | - | - | 453- CGTCA |
| gibberellin-responsive element | - | - | - | - | - | - | - | 691-TCTGTTG |
| MBSI | MYB binding site involved in flavonoid biosynthetic genes regulation | - | - | - | - | - | - | 420- TTTTTACGGTTA |

BgSABP2:No Amplification
